# Ferroelectric Topological Defects in Hexagonal Manganites

**DOI:** 10.3390/ma19010031

**Published:** 2025-12-21

**Authors:** Ziyan Gao, Sang-Wook Cheong, Xueyun Wang

**Affiliations:** 1 College of Science, China Agricultural University, Beijing 100083, China; gaozy@cau.edu.cn; 2 Rutgers Center for Emergent Materials, Rutgers University, Piscataway, NJ 08854, USA; sangc@physics.rutgers.edu; 3 School of Aerospace Engineering & State Key Laboratory of Environment Characteristics and Effects for Near-Space, Beijing Institute of Technology, Beijing 100081, China

**Keywords:** hexagonal manganite, topological defect, domain and domain wall, ferroelectric

## Abstract

Hexagonal rare-earth manganites, as prototypical improper ferroelectrics in which structural distortions give rise to ferroelectricity, exhibit unique physical phenomena that are absent in conventional proper ferroelectrics. Owing to their Z_2_ × Z_3_ topologically protected ferroelectric domain structure, characterized by the convergence of six domains at vortex core, hexagonal manganites can host charged domain walls exhibiting multiple distinct conductive states and unconventional physical effects such as the half-wave rectification effect within a single bulk single crystal, opening up promising avenues for the practical applications. Moreover, as an excellent experimental platform for verifying the Kibble–Zurek mechanism, hexagonal manganites not only possess a broad application potential but also embody rich and fundamental physical insights. Given a series of recent advances in this field, it is essential to systematically summarize and discuss the key findings, current progress, and future research perspectives concerning the hexagonal manganite system. In this review, the origin of ferroelectricity in hexagonal manganites are first clarified, followed by a discussion of the formation and transformation mechanisms of unique ferroelectric domain structures, as well as the intrinsic mechanical properties. Subsequently, the manipulation of topological defects are compared, including electric fields, thermal treatment, oxygen vacancies, and stress–strain fields. Building upon these discussions, the distinct physical effects observed in hexagonal manganites are comprehensively summarized, such as domain wall conductance, dielectric and ferroelectric properties, and thermal conductivity. Finally, based on a detailed summary of the major achievements, the unresolved issues that warrant further investigation are highlighted, thereby offering guidance for future research directions and providing valuable insights for the broader study of ferroelectric materials.

## 1. Introduction

In recent years, topological defects have emerged as a concept of great interest in the fields of condensed matter physics and materials science. Topological defects refer to singular regions in a field distribution; in ferroic materials, they are typically described as features of the order parameter that cannot be eliminated through continuous deformation. Various forms of topological defects, ranging from those existing in the cosmos to those in micro- and nanoscale systems, play a crucial role in shaping a wide array of physical phenomena [1,2]. Examples include the formation of matter states during the Big Bang, tornadoes in the Earth’s atmosphere, vortices on the ocean surface, cell compression and apoptosis in biological systems [3], structural design of mechanical metamaterials [4], screw dislocations and crack propagation in crystals [5], as well as domain structure defects (such as vortex domains) and topological spin textures [6]. In spontaneous symmetry-breaking processes, topological defect serves as a key indicator for tracing the evolution of symmetry, interaction range, and order parameters in condensed matter systems.

Topological defect in ferroelectric materials serve as a crucial carrier for investigating the symmetry and order-parameter evolution of ferroelectric systems [7]. Typically, these topological defects manifest in various forms, such as ferroelectric domains and domain walls formed by regions of differing polarization orientations [8,9], polarization vortices [10,11,12,13], polarization bubbles [14,15], skyrmions [16,17,18,19], and merons [20]. Because electric dipoles of different orientations are coupled with the crystal lattice, ferroelectric topological defects establish a direct connection between real-space topology and emergent physical phenomena, giving rise to an intriguing research field known as topotronics [21]. Due to the competition among gradient, electrostatic, and elastic energies, periodic topological structures have been observed in superlattices such as PbTiO_3_/SrTiO_3_ [22] and Hf_0_._5_Zr_0_._5_O_2_ [23], leading to the emergence of novel phenomena such as stable negative capacitance [24]. With further reduction in system dimensionality, ferroelectric materials exhibit a wealth of topological defects, including polarization bubbles [25] and labyrinthine domains [26] in Pb(Zr,Ti)O_3_ (PZT) thin films, polarization bubbles in CuInP_2_S_6_ [27,28,29,30], as well as ripples and vortex configurations discovered in two-dimensional twisted ferroelectric heterostructures [31,32]. Under the constraints of size effects and boundary conditions, various ferroelectric topological defects, such as quasi-one-dimensional conductive channels [33] and polar Solomon rings [34], have been identified in BiFeO_3_ nanoislands, demonstrating potential applications in logic circuits [35], non-volatile memristors [36], and reconfigurable optical devices [37]. Generally, ferroelectric topological defects confined by material clamping or defect pinning exhibit electrical topological protection, which allows them to remain stable within ferroelectric systems and resist external field perturbations. In contrast to conventional ferroelectric domain structures, which can be readily modulated by conjugate fields (electric fields), ferroelectric topological defects usually require non-conjugate external stimuli for manipulation. This poses a significant challenge for their integration into practical devices. Consequently, achieving localized and precise external-field control over ferroelectric topological defects of different configurations has become a central focus and challenge in current research.

Distinct from ferroelectric heterostructures, nanoislands, low-dimensional thin films, and twisted two-dimensional stacks, the ferroelectric topological defects (vortex cores within vortex domains) in hexagonal manganites *h*-RMnO_3_ (R = Y, Ho-Lu) are thermodynamically, and spontaneously formed in bulk single crystals, a discovery that has attracted extensive research attention since its inception [38]. Hexagonal manganites are improper ferroelectrics whose polarization originates from geometric ferroelectricity. The formation of ferroelectric topological defects in these materials is constrained by the lattice mismatch between the MnO_5_ polyhedra and the rare-earth R ions. During the structural phase transition from the paraelectric P6_3_/mmc phase to the ferroelectric P6_3_cm phase, six ferroelectric domains spontaneously converge at a single point (the vortex core), giving rise to a Z_2_ × Z_3_ vortex domain structure [39]. Experimental studies have revealed that three types of conductive charged domain walls coexist near the vortex cores on the side surfaces of single crystals, offering potential applications in non-volatile multistate memory devices. Moreover, ferroelectric topological defects in hexagonal manganites exhibit a range of exotic physical phenomena, including high carrier mobility, half-wave rectification, AC conduction behavior, and an “insulator-to-conductor” transition modulated by oxygen vacancies. These properties render *h*-RMnO_3_-based systems highly promising for next-generation micro- and nanoelectronic devices. 

Therefore, controllable ferroelectric topological defects in hexagonal manganites holds significant scientific value for advancing the understanding of ferroelectric domain physics and the development of micro/nanoelectronic technologies. Previous reviews on hexagonal manganites have primarily focused on magnetic structures [5] and magnetoelectric coupling [6]. In recent years, a growing body of research has reported numerous important findings regarding topological defects in this system. These studies have not only deepened the understanding of the underlying physical mechanisms and advanced the broader field of ferroelectric topological defects but have also opened new avenues for the application of hexagonal manganites in micro- and nanoelectronic devices. Nevertheless, a comprehensive and systematic discussion on this topic remains lacking. Accordingly, this review focuses on recent progress on experimental advances and does not cover studies based on DFT calculations or phase-field simulations. It mainly concerns the formation and observation of ferroelectric domains and domain walls, multiphysical-field control, emergent physical mechanisms, and macroscopic properties in hexagonal manganites.

## 2. The Origin of Ferroelectricity

### 2.1. Crystal Structure

**Figure 1:**
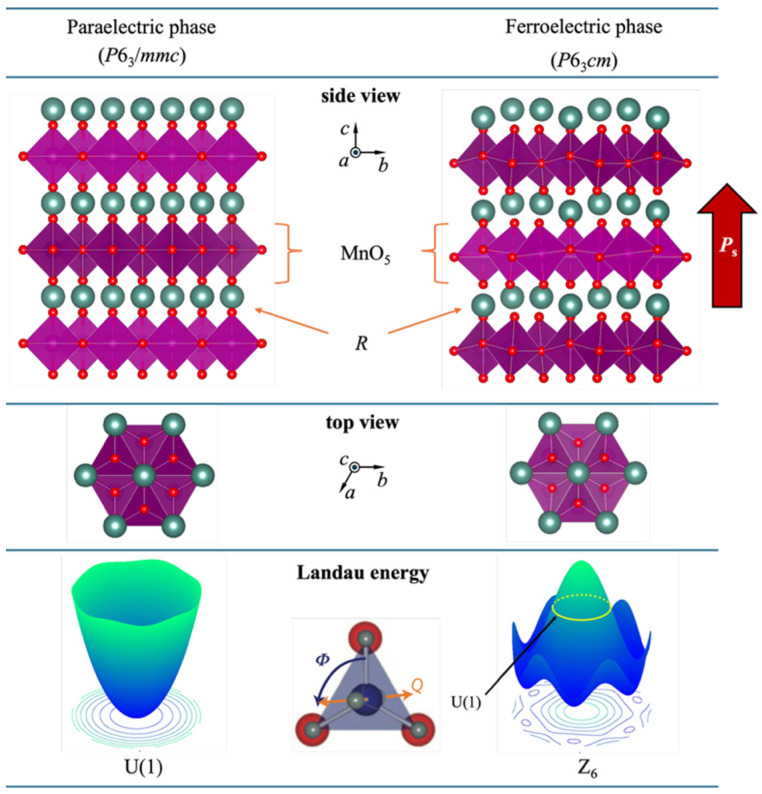
The crystal structure and landau energy of *h*-RMnO_3_ [48,49].

The rare-earth hexagonal manganites can crystallize in either orthorhombic *o*-RMnO_3_ (R = La-Dy) or hexagonal *h*-RMnO_3_ (R = Y, Ho-Lu) systems depending on the rare-earth ions, where the hexagonal crystal structure becomes more stable for rare-earth elements with smaller ionic radii. As an improper ferroelectric material (where the primary order parameters are the structural factor amplitude *Q* and tilting angle *φ*), *h*-RMnO_3_ derives its ferroelectricity from structural distortions. The ferroelectric phase transition temperature (Curie temperature, *T*_C_) ranges from 980 to 1435 °C, depending on the specific R^3+^ ion [40]. The crystal structure of *h*-RMnO_3_ consists of alternating layers of corner-sharing MnO_5_ trigonal bipyramids and R^3+^ ions, as illustrated in Figure 1. In the high-temperature paraelectric phase, all MnO_5_ trigonal bipyramids align along the *c*-axis, while the R^3+^ ion layers are ordered within the *ab*-plane. When *h*-RMnO_3_ crystals are cooled through the Curie point, their crystal structure undergoes a phase transition from the paraelectric phase *P*6_3_/*mmc* to ferroelectric phase *P*6_3_*cm* [41]. In the ferroelectric phase, the MnO_5_ trigonal bipyramids exhibit cooperative tilting in groups of three, either toward or away from the central R^3+^ ion, resulting in a tripling of the unit cell (trimerization). This trimerization occurs around three distinct R^3+^ ions, giving rise to three different trimerized antiphase domains (denoted as a, b, and γ), which differ from each other by 1/3 d or 2/3 d of the lattice (where d represents the in-plane lattice parameter of the newly formed unit cell), referred to as the K_3_ mode [42]. In this K_3_ mode, the tilting of MnO_5_ bipyramids induces a wave-like displacement of R^3+^ ions along the *c*-axis in a 2:1 ratio upward and downward, as illustrated in the side view of Figure 1. While the K_3_ mode confined to the *ab*-plane does not contribute to spontaneous polarization, the emergence of the additional $\Gamma_{2}^{-}$ mode plays a critical role in generating out-of-plane spontaneous polarization (4.5~5.6 μC/cm^2^) [43,44,45,46,47], along the *c*-axis. This polarization is primarily associated with the relative displacement of R^3+^ ions along the +*c* direction and O^2−^ ions along the -*c* direction. When a sufficiently large R^3+^ ionic radius destabilizes the hexagonal structure, favoring a transition to the orthorhombic phase (*o*-RMnO_3_). Thus, the ferroelectricity in *h*-RMnO_3_ originates from the size mismatch between the R^3+^ ions and the MnO_5_ trigonal bipyramids, where the primary order parameters are not the polarization *P* but rather the amplitude *Q* and phase *Φ* of the trimerization-related K_3_ mode. From an energetic perspective, the paraelectric phase corresponds to a potential energy surface exhibiting U(1) symmetry, whereas the ferroelectric phase corresponds to a potential landscape with sixfold Z_6_ symmetry, characterized by a “Mexican-hat”-shaped profile [48]. The brim of the hat represents six degenerate low-energy ground states associated with the phase sequence (α^+^, β^−^, γ^+^, α^−^, β^+^, γ^−^), as illustrated in Figure 1.

### 2.2. Debate of Phase Transition

Since the discovery of the hexagonal manganites in 1963, it has remained a debate of ferroelectric phase transition about single phase transition or two phases transition. It was not until 2015 that Martin Lilienblum et al. revealed that it was a single phase transition process with ferroelectricity determined by topology rather than electrostatics through nonlinear optical experiments and piezoresponse force microscopy, complemented by Monte Carlo simulations [49]. Take YMnO_3_ as an example, dilatometric measurements on the sample reveal a transition temperature of 1266 ± 5 K. The SHG amplitude shows a gradual and continuous decrease towards this value. This temperature dependence is strikingly different from that extrapolated from other measurement and there is no sign of any secondary anomaly. However, the Monte Carlo simulation shows that the amplitude *Q* and phase *Φ*_6_ of the first order parameter have two different transition temperatures *T*_C_^*^ and *T*_C_, which are not consistent with the *T*_C_ of the second order parameter polarization. Therefore, the special structural phase transition of improper ferroelectric hexagonal manganite makes the experimental observation of phase transition temperature misleading. Even though the actual phase transition temperature is *T*_C_, an experimental probe may therefore give an additional false transition at *T*_C_^*^. This is mirrored by the SHG measurement, because SHG probes *P*_s_ directly. This explains perfectly the long-standing “two-phase-transition controversy” and the apparent discrepancy between neutron diffraction experiments and the present SHG results. The same results were also confirmed in ErMnO_3_ [50].

## 3. Intrinsic Mechanical Properties

Investigating and determining the intrinsic mechanical properties of hexagonal manganites is of great significance for understanding the relationship between the microscopic domain structure and the macroscopic ferroelectricity. On the one hand, the ferroelectricity of hexagonal manganites originates from structural distortion. Therefore, it is intrinsically coupled with the stress–strain field. On the other hand, as will be shown in subsequent chapters, mechanical stimuli represent the most effective means of manipulating the ferroelectric domain structures in hexagonal manganites. This chapter summarizes the intrinsic mechanical properties of the hexagonal manganite system, including Young’s modulus, hardness, and fracture toughness, as well as an exploration of the anomalous mechanical behaviors observed during the experiments.

### 3.1. Young’s Modulus

#### 3.1.1. Characterization

**Figure 2:**
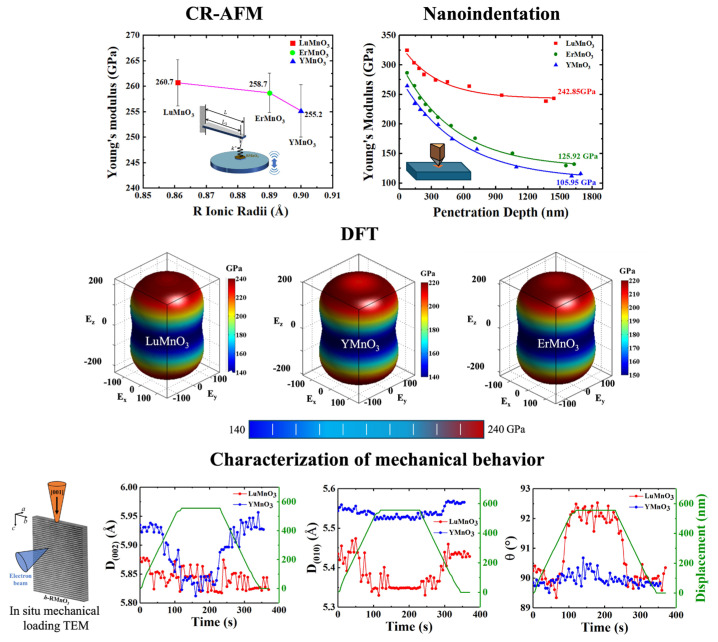
Young’s modulus and anomalous mechanical behavior of hexagonal manganites [55].

The Young’s modulus quantifies the stiffness of a material under longitudinal tension or compression, defined as the ratio between the applied normal stress (force per unit area) and the resulting strain (axial deformation over the original length). It directly reflects a material’s ability to resist deformation within its elastic regime. Notably, previously reported values of the Young’s modulus for YMnO_3_ and ErMnO_3_ ceramics were anomalously low (approximately 20 GPa, which is similar to soft CuInP_2_S_6_) [51,52], significantly smaller than those of typical perovskite oxides, such as SrTiO_3_ (283 GPa) [53]. Yen et al. investigated the Young’s modulus of HoMnO_3_ thin films using nanoindentation techniques, giving a value of 219.2 GPa [54]. However, the measurements were inevitably affected by substrate-induced artifacts. More recently, Gao et al. employed a combination of nanoindentation, contact-resonance atomic force microscopy (CR-AFM), and density functional theory (DFT) calculations on bulk single crystals to determine the intrinsic Young’s modulus of *h*-RMnO_3_, as illustrated in Figure 2 [55].

Due to the distinct measurement principles and operational modes of different characterization techniques, the resulting Young’s modulus values obtained by various methods carry different physical meanings. Since the CR-AFM method determines the modulus based on the contact stiffness between the probe and the sample, it primarily reflects the surface Young’s modulus of the material. Under relatively small applied loads (e.g., 2 mN), the indenter penetrates only shallowly into the sample, and thus the measured Young’s modulus closely matches that obtained from CR-AFM. At sufficiently large loads (e.g., 450 mN), the indentation depth increases, and the modulus–depth curve gradually stabilizes and becomes independent of the indenter geometry. The corresponding Young’s modulus at this stage represents the intrinsic Young’s modulus, which is in closer agreement with the DFT-calculated value. DFT calculations possess a distinct advantage in this context. It not only provides intrinsic Young’s modulus values but also yield the three-dimensional spatial distribution of the modulus. The results reveal that the out-of-plane Young’s modulus along the (001) direction of hexagonal manganites is greater than the in-plane modulus, which originates from the pronounced anisotropy of their crystal lattice structure.

#### 3.1.2. Abnormal Mechanical Behavior

It is noteworthy that the experimentally measured values of ErMnO_3_ and YMnO_3_ deviate significantly from the theoretically calculated ones, particularly as the Young’s modulus exhibits pronounced variations with indentation depth (Figure 2). To elucidate the underlying mechanism, in situ transmission electron microscopy (TEM) experiments were conducted on LuMnO_3_ and YMnO_3_ single crystals to investigate their distinct microscopic mechanical behaviors under applied mechanical loading. A diamond indenter with a diameter of approximately 20 nm was used to apply compressive loads perpendicular to the (001) plane, as illustrated in Figure 2. The results reveal that the (002) interplanar spacing of YMnO_3_ undergoes a substantial contraction (approximately 2%, comparable to the strain magnitude observed in freestanding BaTiO_3_ thin films [56]), whereas that of LuMnO_3_ remains nearly unchanged (a contraction of only 0.2%). When compressed along the (001) direction, YMnO_3_ primarily accommodates mechanical load through the (010) interlayer spacing reduction, resulting in a relatively smaller macroscopic Young’s modulus. In contrast, LuMnO_3_ releases the applied stress via crystallographic plane rotation, where the contraction of the (010) plane contributes predominantly to load bearing, while the (002) spacing experiences only minor change. Consequently, LuMnO_3_ exhibits a load-insensitive and relatively large Young’s modulus along the (001) direction, whereas the modulus of YMnO_3_ (or ErMnO_3_) decreases rapidly with increasing indentation depth.

In terms of mechanical properties, ErMnO_3_ and YMnO_3_ exhibit pronounced differences from LuMnO_3_, which can be attributed to lattice instability. In the RMnO_3_ series containing rare-earth elements with relatively large ionic radii (*R* = La to Dy), the orthorhombic phase is thermodynamically stable. As the ionic radius of R^3+^ decreases (e.g., *R* = Ho to Lu), the hexagonal *P*6_3_*cm* phase becomes increasingly favored [57]. Among all hexagonal manganite crystals, Lu^3+^ (ionic radius 0.861 Å) possesses the smallest ionic radius, and calculations based on the tolerance factor indicate that LuMnO_3_ represents the least stable structure within the hexagonal manganite family [58]. In contrast, the ionic radii of Er^3+^ (0.89 Å) and Y^3+^ (0.90 Å) are comparatively larger. Given that LuMnO_3_ is structurally less stable than ErMnO_3_ and YMnO_3_, it is more susceptible to structural distortion under external mechanical loading. The rotational behavior of LuMnO_3_ observed in in situ mechanical experiments further corroborates this interpretation. Consequently, these factors collectively account for the markedly distinct mechanical behaviors exhibited by ErMnO_3_ and YMnO_3_ compared with LuMnO_3_.

### 3.2. Hardness

Hardness is defined as a material’s ability to resist localized plastic deformation, particularly indentation, scratching, or wear. It is not an intrinsic or absolute physical property, as its value depends on the specific testing method and conditions employed. The hardness *H* of a material can be obtained from nanoindentation experiments as follows:

(1) $$H\;=\;\frac{P}{A}$$

Here, *P* denotes the instantaneous load at a given indentation depth. *A* represents the projected contact area between the indenter and the sample surface. As the indentation depth increases, the hardness of the (001) plane of the hexagonal manganite single crystals gradually decreases and eventually approaches a stable value, which corresponds to the intrinsic hardness of the crystal. The hardness values of the (001) planes for different hexagonal manganite single crystals are listed in Table 1.

### 3.3. Fracture Toughness

Fracture toughness is a quantitative measure of a material’s ability to resist the unstable propagation of macroscopic cracks and serves as a key indicator for evaluating the material’s toughness. The fracture toughness *k* of a material can be determined through nanoindentation experiments, and its calculation is given by the following equation:

(2) $$\;k\;=\;\beta {\left(\frac{E}{H}\right)}^{\frac{1}{2}}P_{\max}L^{-\frac{3}{2}}$$

Here, *β* is an empirical constant related to the indenter geometry. *E* is the elastic modulus. *H* is the hardness. *P_max_* is the peak load. *L* is the length of the radial cracks generated around the indentation. The results are summarized in Table 1, showing that the fracture toughness of the single crystals gradually decreases with increasing ionic radius of the rare-earth element R.

## 4. Ferroelectric Domain and Domain Wall

### 4.1. Unique Ferroelectric Domain Configuration

**Figure 3:**
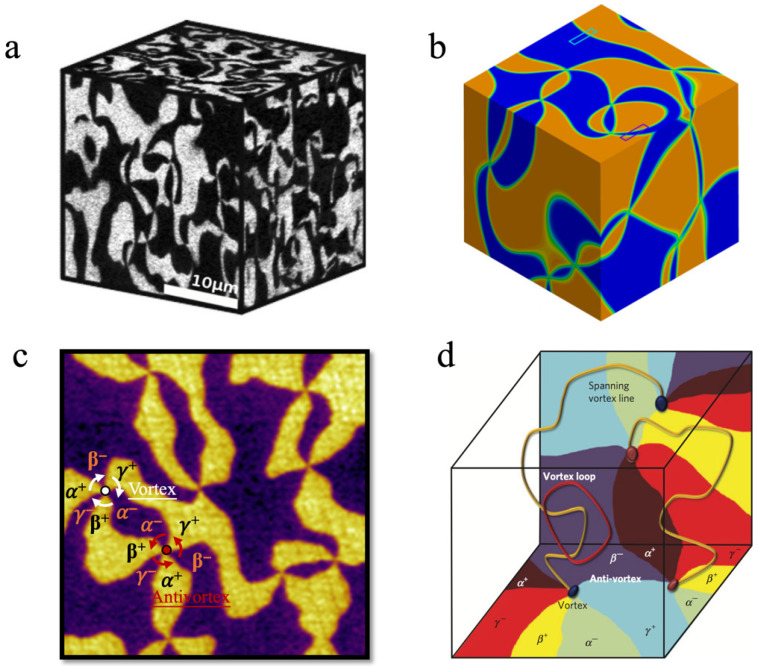
Vortex domain configuration in *h*-RMnO_3_ [2,21,62,63]. (**a**) Three-dimensional distribution of vortex domain structures on experimental; (**b**) Three-dimensional distribution of vortex domain structures on phase-field simulations. The two different colors correspond to ferroelectric domains with opposite polarization orientations, specifically along the positive and negative directions of the *c* axis, respectively.; (**c**) Z_2_ × Z_3_ vortex domains and vortex–antivortex pairs; (**d**) Distribution of (anti)vortex cores within *h*-RMnO_3_ single crystals. The yellow lines are spanning vortex lines.

A wave-like configuration of R^3+^ ions results in a net polarization along the *c*-axis, which in turn gives rise to ferroelectric domains polarized upward or downward. However, early researchers regarded *h*-RMnO_3_ as a uniaxial ferroelectric, and the island-like ferroelectric domains observed due to technical limitations did not attract significant attention [59]. It was not until TEM and PFM characterizations that confirmed the ferroelectric domain structure in *h*-RMnO_3_ consists of six domains converging at a single point with alternating polarization directions [60,61]. The 3D domain structure is isotropic, uniformly and randomly distributed in all orientations, in spite of the layered crystal structure [62,63] (Figure 3a,b). The domain structure can be considered as Z_2_ × Z_3_ vortex domains (clockwise sequence α^+^, β^−^, γ^+^, α^−^, β^+^, γ^−^) and antivortex domains (counterclockwise sequence α^+^, γ^−^, β^+^, α^−^, γ^+^, β^−^), formed by the combination of the K_3_ mode (α, β, γ) and the $\Gamma_{2}^{-}$ mode with opposite polarizations (+, –), in which their convergence points refer to as vortex and antivortex cores, respectively [39,46] (Figure 3c). Layer-by-layer in situ polishing PFM experiments confirm that vortex and antivortex cores are continuously connected throughout the single crystal and terminate at the crystal boundaries [2], shown as Figure 3d. In this case, a reversible long-range motion of domain walls was found in hexagonal manganites [64], which is unique and over much larger distances than commonly expected for ferroelectric systems in their pristine state. Recently, a novel three-dimensional imaging approach of domain and domain wall structures has been demonstrated in a single YMnO_3_ nanocrystal through multi-peak Bragg coherent X-ray diffraction imaging [65]. Such a unique domain structure, combined with the uniaxial polarization along the *c*-axis, gives rise to a variety of intriguing physical phenomena and opens broad prospects for functional applications of *h*-RMnO_3_.

**Figure 4:**
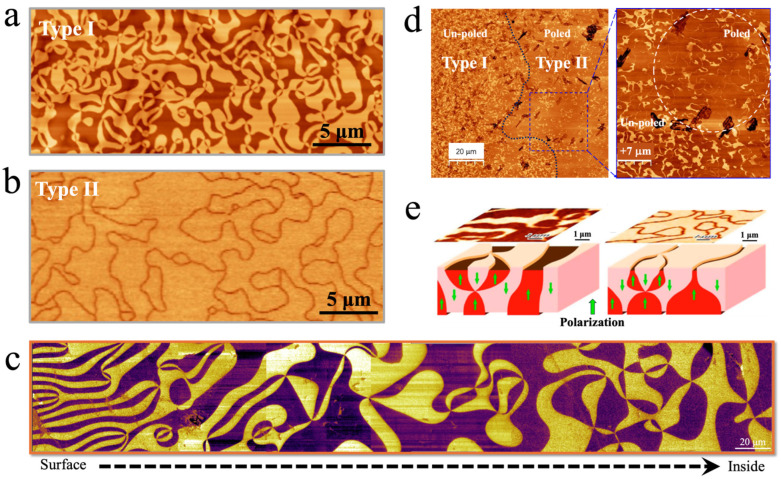
Type-I and type-II domain configurations of vortex domain [70,71,72,74]. (**a**) Type-I domain configuration after annealing in O_2_; (**b**) Type-II domain configuration at the surface of the single crystal; (**c**) Gradual transition from type-II to type-I domain configurations evolving from surface to inside of the single crystal; (**d**) Converts type-I domains into type-II domains by electric poling; (**e**) Domain structures regulated by oxygen defects self-poling effect.

Due to the self-poling effect, originating from oxygen off-stoichiometry near the surface [66], upward and downward polarized domains exhibit an inhomogeneous distribution. Owing to their *p*-type semiconducting nature [67,68,69], *h*-RMnO_3_ naturally favor the formation of narrow upward-polarized and broad downward-polarized domains at the surface of single crystals. However, after annealing in an oxygen atmosphere, this inhomogeneous distribution of oppositely polarized domains disappears, and the domain widths for both polarization directions become nearly identical. The two distinct surface-near vortex domain configurations are classified as type-II and type-I, respectively [70] (Figure 4a,b). This transition from type-II to type-I has also been observed in experimental characterizations of the three-dimensional continuous distribution of vortex domains [71] (Figure 4c). In addition to controlling oxygen stoichiometry to modulate the transition from type-II to type-I, electric poling can also induce the transformation between these two domain configurations, which are associated with the breaking and restoring of the Z_2_ part of the Z_2_ × Z_3_ symmetry [72] (Figure 4d,e). Compared to self-poling, electric poling induces a less thorough transformation of the domain configuration, as unfavored broad domains can still be observed within the type-II domain area [73]. A comprehensive understanding of self-poling induced by oxygen off-stoichiometry and the origin of type-I and type-II vortex domain states in *h*-RMnO_3_ can be found in the dedicated study [74]. Interestingly, these domain configurations in *h*-RMnO_3_ also serve as an excellent platform for validating graph theory, reflecting the intrinsic self-organizing nature of complex phenomena [70,73].

### 4.2. Domain Transformation and Spatial Distribution

**Figure 5:**
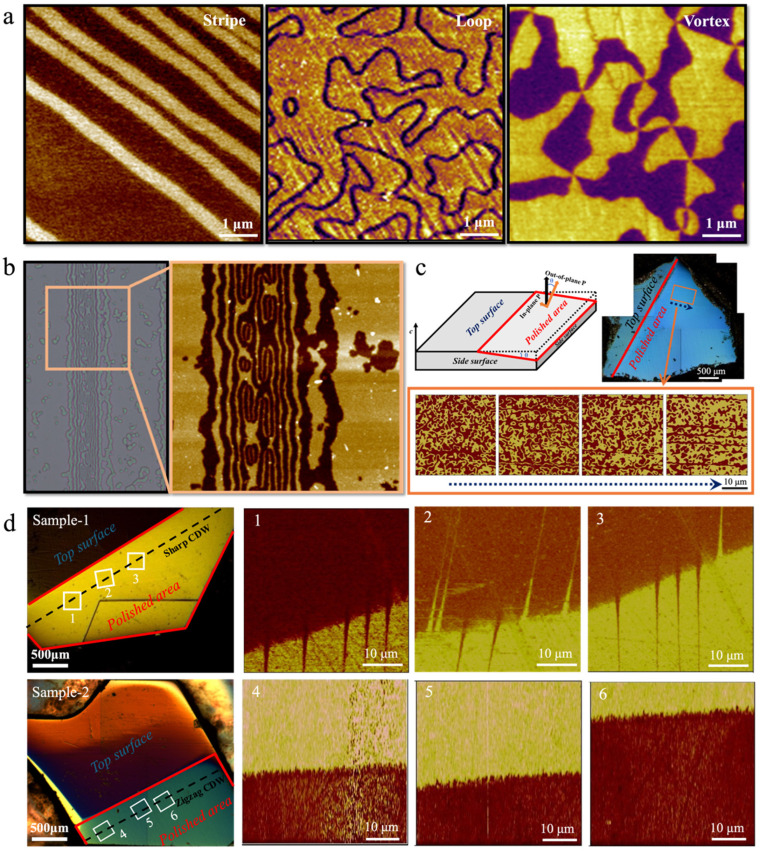
Different domain configurations and their transitions [71,77]. (**a**) Stripe, loop and vortex domain; (**b**) the origin of vortex domain; (**c**) Spatial evolution of loop domains from surface to interior The interrupted arrow indicate the arrangement direction of the scanned images; (**d**) Spatial evolution of stripe domains from surface to interior.

In addition to the Type I and Type II vortex domain structures, *h*-RMnO_3_ also exhibits two other domain configurations: stripes and loops, as shown in Figure 5a. The formation of different domain configurations is closely related to the crystal growth temperature and subsequent thermal treatments [75,76]. Without external perturbations, stripe domains spontaneously form in single crystals grown at temperatures below its ferroelectric transition temperature *T*_C_. Therefore, stripe domains are commonly observed in most as-grown *h*-RMnO_3_ single crystals, except for YMnO_3_, whose *T*_C_ exceeds the growth temperature [75]. Upon annealing at higher temperatures but below *T*_C_, as-grown *h*-RMnO_3_ single crystals develop loop domains [76]. The dynamical formation of vortex domains can be described by the Kibble–Zurek mechanism, which accounts for the non-equilibrium dynamics and the generation of topological defects during continuous phase transitions [2,38].

An elegant approach is to accurately control the annealing temperature at around *T*_C_, thereby capturing the coexistence of vortices, loops, and stripe domains [77]. By analyzing the domain arrangements of vortex–antivortex pairs, the merging process between vortex and stripe domains was identified, revealing that vortex domains are initially formed within narrow stripe domain regions (Figure 5b) [77,78]. Moreover, a refined inclined polishing technique has proven effective in revealing the continuous variation of domain structures from sample surfaces to inside with one scanning PFM on the polished surface (Figure 5c) [71]. This technique provides direct three-dimensional insight into the continuous evolution of loop, stripe, and vortex domains within the crystal. Firstly, this three-dimensional continuous characterization reveals that loop domains progressively evolve into bubble domains, coinciding with a shift in the dominant polarization orientation (Figure 5c). Additionally, the observation of subsurface stripe domains implies that, under high-temperature annealing, loop and bubble domains are likely created around stripe domains and eventually merged with them, providing insights into the domain evolution pathways. However, in as-grown *h*-RMnO_3_ single crystals with stripe domains, the surface stripe domains gradually broaden as they extend into the interior and ultimately stop at a tail-to-tail charged domain wall. Surprisingly, the internal charged domain wall is straight and continuous throughout the crystal (Figure 5d), which is notably distinct from the commonly curved domain walls observed near vortex cores on the side surface containing the *c*-axis in this system [79,80,81,82,83,84]. Since charged domain walls are energetically costly and intrinsically unstable, most naturally occurring charged domain walls in ferroelectrics are curved, as observed in systems such as BiFeO_3_ [85,86], PbTiO_3_ [87,88], BaTiO_3_ [89], (Ca,Sr)_3_Ti_2_O_7_ [90] and others, in order to minimize electrostatic energy and attain a more stable configuration. In as-grown *h*-RMnO_3_ single crystals, the unusual stability of a straight internal charged domain wall is sustained by the presence of oppositely polarized wedge-like domains on both sides of the wall, which serve to reduce the electrostatic energy. In rare cases where no stripe domains are present on the crystal surface, the straight charged domain wall in the interior reduces its electrostatic energy through roughening, which serves as an alternative stabilization mechanism. For vortex domains, a transition from type-I to type-II domain configurations occurs from the surface toward the interior, which originates from the self-poling effect on the crystal surface (Figure 4c). Moreover, the vortex domains are continuously and randomly distributed in three dimensions, extending from the surface into the bulk of the crystal.

### 4.3. Observation Technologies of Ferroelectric Domain and Domain Wall

**Figure 6:**
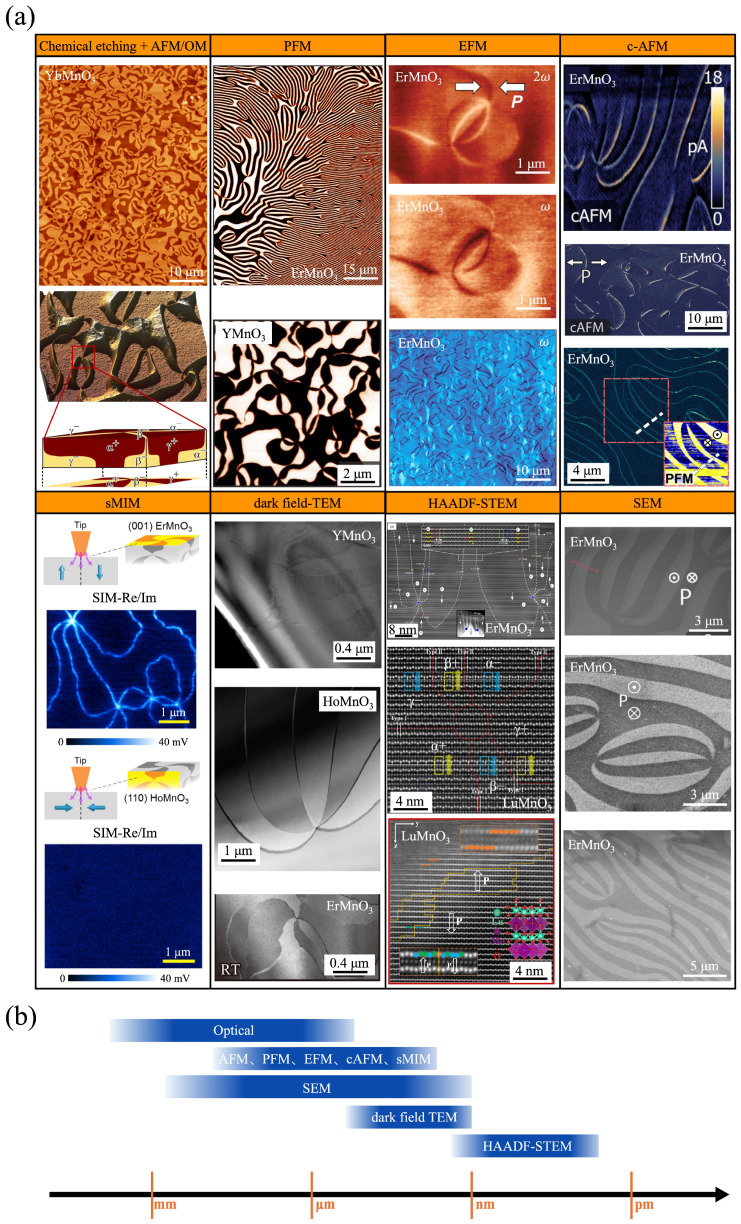
Ferroelectric domains and domain walls characterized by different techniques [21,39,40,46,72,74,75,78,83,99,100,101,106,108,116]. (**a**) Ferroelectric domain structures; (**b**) Covered spatial scales. P denotes the polarization, and the corresponding arrows, ⊗ and ⊙ indicate the polarization direction. ω = 37.4 kHz, is the frequency of AC voltage applied to the tip.

A comprehensive understanding of the unique topological defects in *h*-RMnO_3_ relies heavily on the characterization of ferroelectric domains and domain walls. Starting from the earliest technique of chemical etching combined with optical microscopy (OM), the development of advanced scanning probe microscopy (SPM), scanning electron microscopy (SEM), and transmission electron microscopy (TEM) in recent years has significantly deepened our insights into the spatial distribution and distinctive properties of domain structures. Each characterization technique possesses its own advantages in terms of spatial resolution and probing depth, enabling the acquisition of rich information on ferroelectric domains from different perspectives. A detailed comparison and summary of various characterization methods applicable to ferroelectric domains in *h*-RMnO_3_ is provided below, as illustrated in Figure 6 and summarized in Table 2.

#### 4.3.1. Chemical Etching and Optical Microscope/Atomic Force Microscope

To observe the ferroelectric domain of *h*-RMnO_3_, the easiest and the most convenient method to implement is the chemical etching method. After chemical etching, the topography contrast will be formed between different domains and domain walls, due to the surfaces with the positively charged head part of electric polarization are etched faster than those with the negatively charged tail part [70]. Based on the above principles, the chemical etching method needs to consider these three factors, that is, etching agent, etching temperature, etching time. Specifically, the most widely used etchant is phosphoric acid. And etching temperature and time determine the degree of the final surface topography contrast, which depends on different single crystals and the desired effect. Different single crystals have slightly different effects when using the same parameter, which has a negligible effect on domain observation. One of experienced approach is the crystals were etched chemically in phosphoric acid for 30 min at 130–150 °C [2,70,74]. It should be pointed out that the chemical etching method is the prerequisite for the observation of ferroelectric domains by OM or atomic force microscope (AFM) [2,70,74,75,91,92]. In general, after being etched under appropriate conditions, the domain structures can be readily observed using OM immediately following surface cleaning. Compared to other characterization techniques, OM allows for easy imaging of large-area domain patterns, enabling observation of domain structures on a more macroscopic scale. This also facilitates statistical analysis of domain-related features such as vortex core density, domain width, and domain wall length. Etched samples can also be characterized by AFM in tapping mode to acquire domain distribution at smaller length scales. AFM, especially, can obtain high-resolution three-dimensional information on surface domains with height variations, providing insights into the ferroelectric domain etching process, polarization orientation, and oxygen vacancy modulation [70,74].

#### 4.3.2. Piezoresponse Force Microscopy

Piezoresponse Force Microscopy (PFM) is a common and dominant method of characterizing the domain distribution and polarization properties of materials [93]. PFM, derived from contact-mode AFM, applies an AC signal through the tip to probe the piezoelectric deformation of ferroelectric domains with distinct polarization states. Since there are ferroelectric domains with different polarization directions on the surface of *h*-RMnO_3_ single crystal, PFM can be used to observe the contrast changes caused by different polarizations [38,49,76,80,82,83,94,95,96,97]. Based on this principle, PFM characterization imposes relatively simple sample requirements, needing only a flat surface with nanoscale roughness (typically below 100 nm). Therefore, PFM is a versatile, easy-to-handle, and non-invasive technique for imaging ferroelectric domain patterns across a wide range of ferroelectric materials.

#### 4.3.3. Electrostatic Force Microscopy

In contrast to PFM operated in contact mode, Electrostatic Force Microscopy (EFM) is fundamentally based on the non-contact mode of AFM [93], enabling the detection of long-range electrostatic interactions. Due to the adsorption of different surface charges associated with distinct polarization orientations to maintain charge neutrality, EFM can effectively probe the resulting spatial variations in electrostatic potential on the surface of ferroelectric materials. The technique is mostly used on samples that are a mix of conductive and non-conductive areas, which will tend to show contrast where the conductivity changes dramatically. Therefore, EFM based on electrostatic interactions between the probe and the sample surface is suitable for testing charged domain walls where conductivity changes sharply in *h*-RMnO_3_ [47,98,99]. EFM is explicitly sensitive to variations in the density of mobile and fixed charges without unwanted contributions from contact resistance that may obscure the data. Therefore, EFM can reveal that the intrinsic variation in electronic conductivity is not attributed to contact resistance, by detecting local changes in hole density at the domain walls [100]. Recently, the X-ray photoemission electron microscopy (X-PEEM) was applied to detect uncompensated bound-charges at ferroelectric head-to-head and tail-to-tail domain walls in Er_0_._99_Ca_0_._01_MnO_3_ [83]. In comparison to previously applied low-temperature EFM experiments [79], X-PEEM readily distinguishes between positive and negative bound charges, offering high sensitivity and substantially shorter data acquisition times.

#### 4.3.4. Conductive AFM

Conductive atomic force microscopy (cAFM), developed from the contact mode of AFM, enables simultaneous measurement of surface topography and the current flowing through the contact point between the probe and the sample. Compared to EFM, cAFM can quantitatively measure the carrier charge concentrations of domains and domain walls. Usually, the experimental setup requires a complete electric circuit, and then the probe probes the conductivity characteristics of the sample surface. The cAFM technique is very important for observing the DC conductivity of *h*-RMnO_3_ single crystals, especially related to domain wall conductivity [46,80,82,83,84,101], half-wave rectification [81], polarization-modulated rectification [47], moiré patterns [102], etc.

#### 4.3.5. Scanning Microwave Impedance Microscopy

As a new technology based on AFM developed in recent years, scanning microwave impedance microscopy (sMIM) uses microwave frequencies (GHz) AC to measure electrical properties as a localized measurement at the nanoscale. The sMIM process the signal and output the imaginary and real component of the tip-same admittance in sMIM-C and sMIM-R, which correspond to the material permittivity and conductivity. The sMIM technology does not have high requirements on samples, which is consistent with the requirements of AFM, but due to the microwave impedance properties of the test materials, the probes used must have good impedance matching with the samples to be tested [103]. The development of sMIM technology has expanded the in-depth research on AC conductance of *h*-RMnO_3_, involving domain wall movement [103], microwave conductivity [104], etc.

#### 4.3.6. Dark Field TEM

Besides scanning probe techniques, Dark field TEM is another technology that can directly observe *h*-RMnO_3_ single crystal ferroelectric domains. This technology is based on transmission electron imaging under vacuum, so it requires complex sample preparation. Dark-field TEM provides a special perspective to reflect the contrast between different domains to distinguish between different polarization states. Dark-field TEM is commonly used to observe domain switching [105,106], the distribution of vortex–antivortex domains [46,47,74,75,82], and accurately locate the domain wall position for the study of polarization at the atomic scale [105,106,107,108,109,110]. Distinct from the aforementioned scanning probe techniques, Dark field TEM enables the observation of domain structures at a more localized (nanoscale) region, and allows in situ investigation of domain evolution under external stimuli such as electric fields, mechanical stress, electron beam, electric arc heating [105,106,108,111].

#### 4.3.7. HAADF STEM

High-angle annular dark-field (HAADF) imaging is a STEM technique that produces an annular dark-field image formed by very high-angle, incoherent scattered electrons rather than Bragg scattered electrons. This technique is very sensitive to changes in the number of atoms in the sample (*z* contrast image) and can detect stronger signals from atoms with higher *z* values. Therefore, the polarization caused by the offset of the atoms in the z direction of *h*-RMnO_3_ can be observed at the atomic scale. Based on the above principles, HADDF STEM technology provides a very high real spatial resolution for the study of *h*-RMnO_3_ domain structure, including domain polarization direction determination [105,106,107], atomic displacement observation [84,108,112,113], distinguish between domain walls [32,91,110], and strain distribution [21]. Among various characterization methods, HAADF-STEM provides the highest spatial resolution (sub-nanometer levels), and enabling highly localized insights into domain structures, such as the non-polar state at vortex cores [39,114].

#### 4.3.8. Scanning Electron Microscopy

SEM has an outstanding potential for domain and domain wall related investigations, offering contact-free and non-destructive high-speed imaging, nanoscale spatial resolution, and a high flexibility in terms of specimen preparation and geometry that allows, for example, to combine microscopy with nano-structuring or in situ/in operando transport measurements [115]. In contrast to PFM, where image formation relies on difference in the electromechanical response of domains, SEM exploits differences in electron emission yield, allowing high-resolution microscopy experiments without the need for electrical contacts. The imaging rate of SEM is also higher than PFM, which allows capturing the dynamics with better time resolution [116]. Compared to the other methods, SEM sticks out because of the remarkably large range of length scales it can cover (from millimeter to nanometer scale). This correlation between SEM contrast and polarization direction holds if imaging parameters are kept constant and can therefore be used to track changes in the domain structure. A more detailed discussion of SEM domain and domain wall contrast in ferroelectric materials can be found in other comprehensive review article [115]. In recent years, SEM has emerged as one of the unique means of studying *h*-RMnO_3_ domains and domain walls [117,118,119]. Based on secondary electron imaging, SEM enables nanoscale spatial resolution of near-surface domain wall morphology with a probing depth of approximately 1.5 μm. Compared to other techniques with similar resolution (such as 3D imaging by tomographic PFM or FIB), SEM offers non-destructive imaging with rapid acquisition (on the order of seconds), allowing large-area (∼100 × 100 μm^2^) nanoscale mapping in a single scan and providing key parameters such as domain wall curvature and local charge states that are often inaccessible by conventional methods [120].

### 4.4. Duality

The Z_2_ × Z_3_ vortex domains formed in hexagonal manganites originate from the coupling between the trimerization K_3_ mode (tilting of the MnO_5_ trigonal bipyramids) and the $\Gamma_{2}^{-}$ mode (polar displacements of the rare-earth cation sublattice), which collectively stabilize the system energy into six degenerate states. When $\Gamma_{2}^{-}$ is positive, the lowest-energy configurations correspond to K_3_ tilting angles of 0°, 120°, and 240°, whereas for negative $\Gamma_{2}^{-}$, the most stable orientations occur at 60°, 180°, and 300°. The resulting coupling produces a modified “Mexican-hat” potential with six local minima along the brim, corresponding to alternating polarization orientations (see Figure 1). In hexagonal InMnO_3_ (*h*-InMnO_3_), which shares the same ferroelectric structure as *h*-RMnO_3_ compounds, a nearly degenerate and partially undistorted antipolar (PUA) phase (space group $P\overset{ˉ}{3}c$) can be stabilized through rapid quenching. The $P\overset{ˉ}{3}c\;$structure derives from the high-temperature prototype *P*6_3_/*mmc* phase via moderate tilting of the MnO_5_ bipyramids by 30°, 90°, 150°, 210°, 270°, and 330°, accompanied by an “up–down–zero” displacement pattern of the In ions. Symmetry analysis indicates that this PUA state should, in principle, host an antipolar Z_2_ × Z_3_ vortex structure analogous to that of the ferroelectric phase, where the six possible domain states correspond to combinations of three distinct phase shifts (τ, ν, μ) and two oxygen-distortion chiralities. Finally, the existence of these Z_2_ × Z_3_ PUA vortices (τ, ν^∗^, μ, τ^∗^, ν, μ^∗^), which is “dual” to the well-known Z_2_ × Z_3_ ferroelectric vortices (α^+^, β^−^, γ^+^, α^−^, β^+^, γ^−^) in their exchange of ferroelectricity and antipolarity, forming α^+^, τ, β^−^, ν^∗^, γ^+^, μ, α^−^, τ^∗^, β^+^, ν, γ^−^, μ^∗^, vortex domains configuration with a 30° phase difference. However, such antipolar Z_2_ × Z_3_ vortices have not been experimentally observed in quenched *h*-InMnO_3_, likely due to the high density of structural disorder present in the quenched samples [121]. Huang et al. introduced Ga doping at the Mn sites to impose an effective in-plane compressive strain, which has been shown to tune the energy balance between the ferroelectric and antipolar phases, thereby stabilizing the antipolar phase as the ground state. As a result, the antipolar PUA phase was successfully stabilized, and novel antipolar PUA vortex domains were identified. Importantly, this discovery reveals a unique duality between topological antipolar vortices and ferroelectric vortices. The study demonstrates a striking contrast in domain-wall characteristics between the two phases: narrow antipolar domain walls are formed in the ferroelectric phase, whereas wide polar domain walls up to 6 nm in width emerge in the PUA phase [109,110]. These findings unveil an unprecedented intrinsic correlation among topological defect duality, lattice symmetry, and system energetics, opening new avenues for exploring the practical applications of topological defects.

### 4.5. Graph Theory

The self-organizing vortex-domain structure formed in hexagonal manganites can be neatly analyzed by the graph theory. As a branch of mathematics, graph theory has evolved over centuries into a fundamental tool for characterizing the underlying connectivity of complex configurations across disciplines such as mathematics, economics, and computer science. A graph consists of two sets: a non-empty set of vertices (objects) and a set of edges (connections between objects).

**Figure 7:**
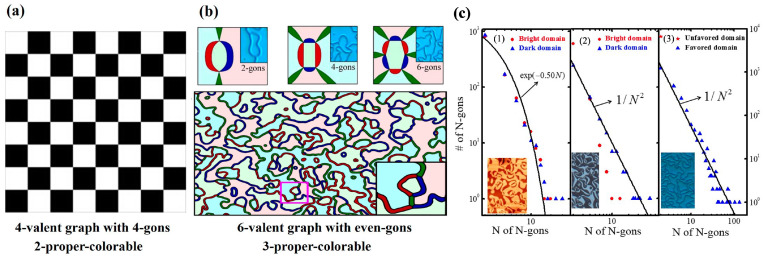
Graph theory in hexagonal manganites [70,73]. (**a**) The checkerboard which is 4-valent graph with 4 gons and 2 proper colorable graph; (**b**) The vortices domain network which is 6-valent graph with even-gons, and it is 3 proper-colorable network; (**c**) N-gon statistical analysis. (**c**): (1) an exponential-law distribution within a type-I network in YbMnO_3_. (**c**): (2) An intermediate network in ErMnO_3_ crystal. (**c**): (3) A power-law behavior within a type-II network in YMnO_3_.

A canonical example in graph theory is the Four-Color Theorem, which states that any two-dimensional map can be colored with no more than four distinct colors such that adjacent regions share no common color, as illustrated in Figure 7a. Each minimal square cell (which may be regarded as a domain) contains four edges and four vertices, forming a four-valent graph composed of quadrilaterals. Because of its inherent periodicity or underlying constraints, such a network can be distinctly colored using only two colors, known in graph theory as a 2-properly colorable graph. Analogously, the vortex-domain network in hexagonal manganites exhibits the characteristics of a six-valent graph, in which each vortex node connects six domain walls, and each domain is enclosed by N (even) vertices or edges, forming an N-gon lattice. Owing to the topological protection of the vortices, the entire domain network can be colored using only three colors, corresponding to a 3-properly colorable graph, as exemplified in Figure 7b for 2-, 4-, and 6-gons configurations. It is noteworthy that in the lower schematic, dark and light shades of blue, green, and red are used to distinguish upward and downward polarization directions. Although six visual colors are shown, only three are mathematically sufficient for complete coloring. Chae et al. provided a rigorous mathematical proof that even-gon six-valent graphs admit a Z_2_ × Z_3_ coloring scheme [72].

Complex networks, such as neural networks, the Internet, and actor collaboration networks, exhibit nontrivial topological characteristics that are absent in simple networks like crystalline lattices. Some complex networks display scale-free power–law degree distributions, whose hallmark features include the presence of a few highly connected nodes (with degrees far exceeding the average) and exceptionally short average path lengths, both of which have attracted widespread attention. Two principal mechanisms have been proposed to account for the emergence of such scale-free distributions: preferential attachment and self-organized criticality. [70]. The underlying physical mechanisms of these vortex-domain networks can be elucidated through N-gon statistical analyses of three representative samples: YbMnO_3_ (type-I network), ErMnO_3_ (intermediate I/II-type network), and YMnO_3_ (type-II network), as illustrated in Figure 7c. The N-gon distribution in type-I networks can be well fitted by an exponential function with a large-N cutoff, indicating an exponential degree distribution in the corresponding dual graph, consistent with observations in other network systems [122,123]. In contrast, the type-II network follows a power–law distribution proportional to 1/N^2^. Networks with such power–law degree distributions are classified as scale-free networks, whose topology has been shown to strongly influence self-organized critical phenomena [124,125]. Notably, during the structural transition from type-II to type-I networks, the slope of the N-gon distribution evolves systematically. In type-II networks, the slope associated with bright (minority) domains gradually increases and eventually converges to a single point, corresponding to the configuration in which all minority domains become two-gons. Phase-field simulations [126] further reveal that type-I networks exhibit a log-normal distribution of N-gons, where the logarithm of the gon count follows a normal distribution, whereas type-II networks maintain a scale-free power–law distribution. The observed transition in distribution patterns is primarily governed by the merging and splitting dynamics of N-gons during the network evolution. Among the relevant mechanisms, preferential attachment (larger gons are more likely to merge with other large gons) has been identified as the fundamental origin of the observed power–law distribution. This finding indicates that preferential attachment plays a dominant role in establishing the scale-free nature of the vortex-domain networks.

## 5. Manipulation of Topological Defects

### 5.1. Electric Field

**Figure 8:**
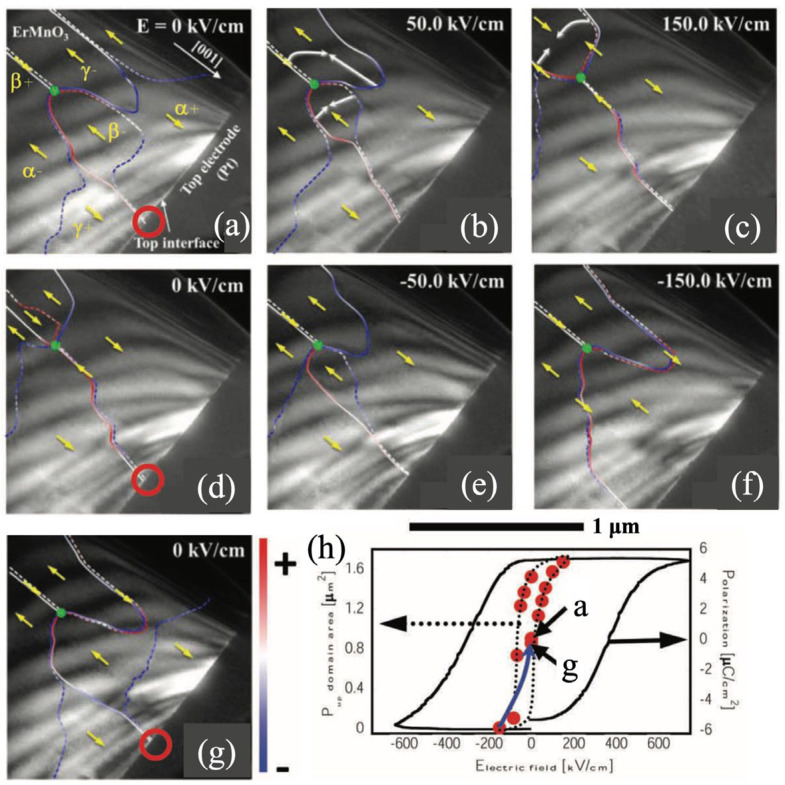
Electric field manipulation of ferroelectric topological defects [105]. (**a**–**g**) Dark-field images showing the order of the switching sequence, denoted alphabetically, with an applied fi eld along the (001) direction. Yellow arrows indicate the polarization direction for each domain. The vortex core is denoted by green dots. Electrostatic charges associated with the domain walls are indicated in red (positive) and blue (negative). The abrupt changes in domain-wall’s position from 50 kV/cm to 66.7 kV/cm, from 150 kV/cm to 0 kV/cm, and from −33.3 kV/cm to −50 kV/cm are shown by white arrows. Note that three 0 kV/cm states have similar configurations of the surface domain, indicated by the red circles in (**a**,**d**,**g**). A hysteresis loop (**h**) was obtained by measuring the P_up_ domain areas for each biased condition represented by red dots. Significant back switching, indicated with the blue arrow (from (**f**–**g**)), is visible. For comparison, a P-E loop electrically measured from a bulk LuMnO_3_ crystal is also shown.

As the conjugate field of ferroelectric polarization, the electric field is the most intuitive and commonly considered means of manipulation. In ferroelectric phases of *h*-RMnO_3_, the polarization vanishes at domain walls, which always converge at a single point to form a vortex core (topological defect). Under an external electric field, domains with polarization aligned to the field direction expand, while those with antiparallel polarization shrink, and the domain walls remain mobile as in conventional ferroelectrics, as shown in Figure 8 [105]. However, due to the neutralized polarization, the vortex cores remain immobile when the polarization reaches saturation, indicating that the external electric field cannot effectively modulate the positions of the topological defects. This conclusion has also been confirmed by multiple in situ TEM experiments under applied electric fields [108,111]. Moreover, under sufficiently high electric fields, the vortex domain structure may be disrupted [74]. Note that the effective manipulation of ferroelectric domain walls while keeping the vortex network complexity has potential application in reservoir computing [127].

### 5.2. Kibble–Zurek Mechanism

Symmetry breaking has not only guided the birth of our universe in cosmological studies but also governs the physical properties of materials in condensed matter physics. On one hand, it is hypothesized that as the universe expanded and cooled from its initial hot and dense state, the vacuum underwent a sequence of phase transitions accompanied by successive symmetry breakings. On the other hand, symmetry breaking in materials gives rise to a wealth of emergent phenomena, such as multiferroicity, magnetoelectric coupling, and skyrmions. These two seemingly disparate fields, cosmology and condensed matter physics, are deeply interconnected through the shared concepts of symmetry breaking and phase transitions. In the realm of cosmology, Kibble first investigated the relationship between the possible topological structures of defects and the breaking of gauge symmetries [128]. Subsequently, Zurek derived a scaling law that relates the density of defects to the quench rate through the critical point [129]. Together, their theories constitute the Kibble–Zurek mechanism (KZM), which describes the nonequilibrium dynamics accompanying symmetry-breaking phase transitions and allows the estimation of topological defect density based on the quench rate. When a system undergoes a nonequilibrium continuous phase transition, the emergence of critical slowing down near the critical point leads to a loss of adiabaticity. The resulting local choice of broken-symmetry states, induced by this critical slowing down, forms the physical origin of topological defect generation.

However, experimental verification of this mechanism is extremely challenging, particularly on cosmological scales where direct observations are practically infeasible. Since the Kibble–Zurek scaling is governed by critical behavior and is expected to be universal across all systems within the same universality class. In condensed matter systems, the KZM describes the nonequilibrium dynamics of second-order phase transitions and predicts a power–law relationship between the cooling rate and the density of topological defects [130]. The main distinction between cosmological and laboratory realizations lies in the fact that, in the latter, the relaxation time and coherence length (the velocity of the relevant sound rather than the speed of light) determine the sonic horizon, denoted by the linear dimension ξ, representing the size of regions that can undergo symmetry breaking coherently. The relationship between the topological defect density *n* and the cooling rate can be expressed as follows:

(3) $$\;n\;=\;\frac{1}{\xi_{0}^{D-d}}{\left(\frac{\tau_{0}}{\tau_{Q}}\right)}^{\left(D-d\right)\frac{z}{1+zv}}$$

where *D* and *d* denote the spatial dimensionality of the system and the dimensionality of the defect, respectively. For example, in a three-dimensional superfluid, a vortex line corresponds to *D* = 3 and *d* = 1, as the defect extends as a one-dimensional curve within the three-dimensional space. The exponents *ν* and *z* represent the spatial and dynamical critical exponents, respectively. *τ*_0_ is a microscopic timescale determined by the intrinsic properties of the system, while *τ*_Q_ denotes the characteristic quench time governing the rate at which the system is driven through the phase transition.

**Figure 9:**
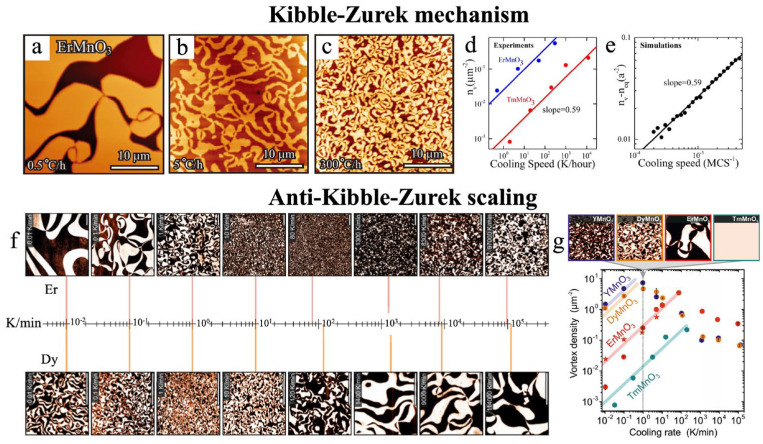
KZM in hexagonal manganites system [2,38,75]. Starlike red symbols indicate data points taken on flux-grown samples. (**a**–**c**) The vortex domains of ErMnO_3_ obtained under different cooling rates during the high-temperature annealing process. (**d**,**e**) The relationship between the topological defect density and the cooling rate, as determined from experiments and Monte Carlo simulations, respectively. (**f**) The vortex domains of ErMnO_3_ and DyMnO_3_ obtained under different cooling rates during the high-temperature annealing process. (**g**) The relationship between the topological defect density and the cooling rate for YMnO_3_, DyMnO_3_, ErMnO_3_, and TmMnO_3_, together with the corresponding vortex domains.

Fortunately, the hexagonal manganite system provides an excellent experimental platform for validating the KZM. When naturally grown single crystals of hexagonal manganites are annealed at temperatures above the *T*_C_ and subsequently cooled down to room temperature, they spontaneously form three-dimensionally distributed topological defects and ferroelectric vortex domain structures. Chae et al. investigated this behavior in ErMnO_3_ single crystals by varying the cooling rate during the annealing process from 0.5 °C/h to 300 °C/h, thereby altering the topological defect density by nearly three orders of magnitude, as shown in Figure 9 [75]. Similar experiments were also performed on TmMnO_3_, YMnO_3_, DyMnO_3_ single crystals [2,38]. The results reveal a linear relationship between the logarithm of the cooling rate and that of the defect density, consistent with the power–law dependence predicted by the KZM. Remarkably, the experimentally determined slope of 0.59 shows excellent agreement with the theoretical prediction. The obtained exponent of 0.59 is very close to the value 2*v*/(1 *+ zv*) ≈ 0.57 that is expected for a 3D *XY* fixed point: *ν* = 0.67155 [131] and *z* ≈ 2 [132].

Moreover, previous studies have reported an unexpected phenomenon known as anti-KZM, referring to a pronounced reversal of the conventional Kibble–Zurek scaling behavior. In this regime, extremely rapid quenches suppress the formation of topological defects, leading instead to an increase in the characteristic size of domain structures, as illustrated in Figure 9 [2,62]. Theoretical investigations have revealed that the KZM inherently encompasses a dynamic crossover from the Ginzburg regime (critical fluctuations dominate and asymptotic scaling prevails) to the mean-field regime (fluctuations act merely as perturbations) [38]. This crossover in scaling behavior is universal, expected to manifest across phase transitions ranging from the atomic to the cosmological scale. By comparing theoretical predictions with experimental data from the hexagonal manganite family, it has been demonstrated that this scaling crossover serves as the intrinsic origin of the observed reversal in the dependence of topological defect density on cooling rate, namely, the anti-Kibble–Zurek scaling law. In addition, temperature-dependent processes associated with chemical and mechanical impurities may also influence the formation of topological defects in RMnO_3_. Experimental observations indicate that even for the same compound, samples from different growth batches, obtained under slightly varied synthesis conditions, can exhibit notable fluctuations in vortex density at a fixed cooling rate. These factors critically affect the thermal conductivity of the crystals, which represents another key parameter governing the overall thermal equilibration of the system. When the thermal conductivity is insufficient, spatial temperature inhomogeneities arise during cooling, leading to asynchronous local phase transitions across the sample. When this velocity is much lower than that of order-parameter fluctuations, defect formation is strongly suppressed. Moreover, this suppression effect tends to vary systematically with chemical composition. As reported by Pang et al., the reduced efficiency of the KZM may be attributed to the nonuniform distribution of local topological defect densities, induced by variations in chemical defect concentrations and surface oxygen vacancies [133].

Beyond serving as a model platform for validating the KZM, hexagonal manganites can also exploit this mechanism as a means of controlling ferroelectric topological domain structures. Compared with other tuning approaches, the use of KZM enables global control over both domain size and domain-wall length across the entire sample. Such control yields distinctive functional effects, for instance, a reduction of approximately 30% in thermal conductivity at room temperature has been achieved in hexagonal manganites through KZM-mediated domain engineering.

### 5.3. Oxygen Deficiency/Interstitial Oxygen

After the single-crystal bulk or thin film undergoes a thermal treatment process, a nonuniform distribution of oxygen vacancy concentration is formed at different depths from the surface, leading to a deviation of ideal stoichiometry. In this manner, the presence of oxygen vacancies alters the surface charge distribution of the material, thereby balancing and compensating for the spontaneous polarization inherent to the ferroelectric material. Therefore, controlling oxygen defects can influence the polarization of ferroelectric materials, enabling the regulation of ferroelectric domain structures. For example, in LiNbO_3_, the gradient of lithium defects leads to the formation of a large number of charged ferroelectric domain walls [134,135,136], and reversible ferroelectric single-domain structures can be induced by varying the oxygen stoichiometry [66]. Similarly, oxygen defects can also modify the ferroelectric domain structures in hexagonal manganite systems.

The formation mechanism of oxygen vacancies in hexagonal manganites can be described as follows: when an *h*-RMnO_3_ crystal is heated to sufficiently high temperatures (above 1000 °C), the ample thermal energy and enhanced ionic diffusivity promote the formation of relatively homogeneous oxygen vacancies throughout the crystal. However, during cooling in an oxygen-containing atmosphere to the temperature range around 700 °C, oxygen atoms begin to diffuse inward. Unless the cooling rate is unrealistically slow, the limited diffusion length constrains the oxygen replenishment to only the near-surface region of the crystal. Consequently, a natural gradient in oxygen vacancy concentration develops from the surface toward the bulk: the surface layer remains nearly stoichiometric with respect to oxygen, whereas the interior of the crystal may still retain a substantial amount of oxygen vacancies.

To investigate the effect of oxygen stoichiometry on domain structures, two HoMnO_3_ single crystals were annealed in an argon atmosphere at temperatures above the *T*_C_ [74]. After annealing, the crystals were slowly cooled to 1000 °C and then rapidly quenched to room temperature. This thermal treatment resulted in a uniform distribution of oxygen vacancies throughout the entire crystal. Consequently, a domain structure consisting of uniformly distributed upward- and downward-polarized ferroelectric domains was formed (type-I domain configuration), as illustrated in Figure 4e. Subsequently, the single crystals were re-annealed in air at 700 °C for 5 h and then furnace-cooled to room temperature. Due to the limited diffusion length of oxygen, this re-annealing process in air generated a gradient of oxygen vacancies along the depth direction. After re-annealing, the crystal surface exhibited a type-Ⅱ domain configuration, characterized by wider downward-polarized domains and narrower upward-polarized domains. Because of the large oxygen-vacancy gradient and the reduced vacancy concentration near the surface, an upward-oriented built-in electric field was established within the crystal, favoring the dominance of upward-polarized ferroelectric domains [70,72,74].

### 5.4. Stress and Strain Fields

**Figure 10:**
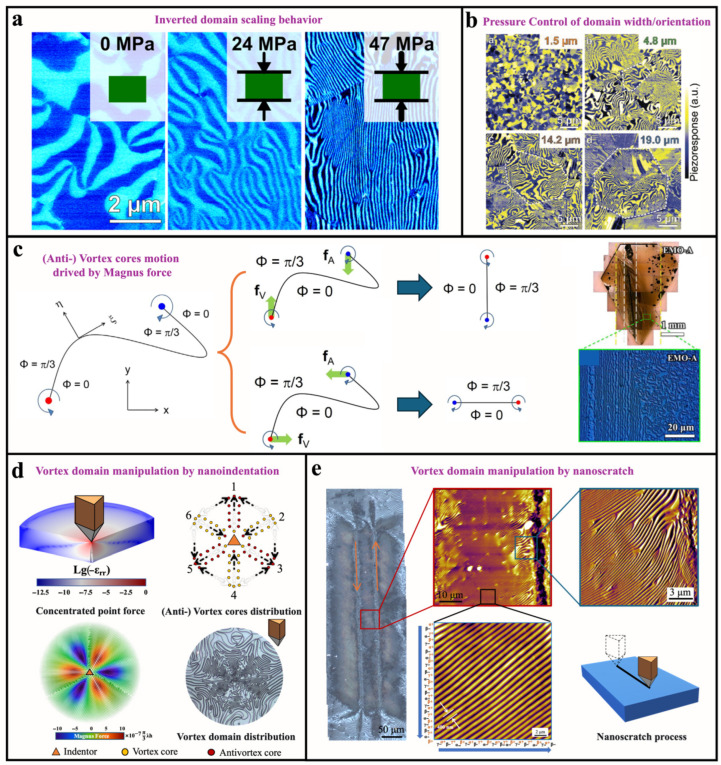
Manipulation of topological defects via stress–strain fields [21,91,137,138]. (**a**) Inverted domain scaling behavior; (**b**) Pressure control of domain width and orientation; (**c**) Vortex and antivortex cores motion-derived by Magnus force. The thin arrows indicate the rotation direction of the phase angle Φ, while the green arrows denote the direction of the Magnus force; (**d**) Vortex domain manipulation by nanoindentation. The black and white arrows represent the Magnus forces acting on the vortex and antivortex cores, respectively; (**e**) Vortex domain manipulation by nanoscratch. The orange arrows indicate the scratching direction.

The elastic strain field profoundly influences the domain structure of hexagonal manganites system. Compared to classical ferroelectrics, an inverted domain size effect is observed in hexagonal manganites: as the grain size increase, smaller domains are formed (Figure 10a) [137]. Under external pressure, the non-ferroelastic ferroelectric domains transform from a random state to an ordered state, and the width of the stripe-like domains depends on the applied pressure (Figure 10b) [138]. Naturally, these phenomena reflect the coupling relationship between the stress–strain field and the polarization.

As an improper ferroelectric system, the ferroelectricity of hexagonal manganites originates from structural distortions involving the rare-earth ions and the MnO_5_ bipyramids. Its primary order parameters are not the polarization *P*, but rather the trimerization amplitude *Q* and phase *Φ*. Furthermore, with increasing temperature up to the Curie point, the *Q* and *Φ* do not exhibit a cooperative reduction that would lead to the disappearance of polarization at the *T*_C_ (Figure 1). This phenomenon initially gave rise to considerable debate regarding the existence of multiple phase-transition temperatures and the precise determination of the Curie point in this system, until the controversy was clarified by Martin et al. in 2015 [49]. Thus, based on the fundamental parameters obtained from DFT calculations, Sergey et al. derived the Landau free energy *f*_L_ and gradient energy *f*_G_ for the hexagonal manganite system [139]. Considering the effect of the applied strain field (*f*_strain_), the system free *F_f_* energy can be expressed as:

(4) $$F_{f}=f_{L}+f_{G}+f_{strain}$$

(5) $$f_{L}=\frac{a}{2}Q^{2}+\frac{b}{4}Q^{4}+\frac{Q^{6}}{6}\left(c+c^{\prime }\cos6\Phi \right)-gQ^{3}P\cos3\Phi +\frac{g^{\prime }}{2}Q^{2}P^{2}+\frac{a_{P}}{2}P^{2}$$

(6) $$f_{G}=\frac{1}{2}\sum_{i=x,y,z}\left[s_{Q}^{i}\left(\partial_{i}Q\partial_{i}Q+Q^{2}\partial_{i}\Phi \partial_{i}\Phi \right)+s_{P}^{i}\partial_{i}P\partial_{i}P\right]$$

(7) $$f_{strain}=-GQ^{2}\left[\left(\varepsilon_{xx}-\varepsilon_{yy}\right)\partial_{x}\Phi -2\varepsilon_{xy}\partial_{y}\Phi \right]$$

where *Q* and *Φ*, are the amplitude and phase of the trimerization mode (K_3_). *P* is the local amplitude of polar mode $\Gamma_{2}^{-}$). The subscripts (*x*, *y*, *z*) are the major coordinate axes. *a*, *b*, and *c* are the constants for the free-energy polynomial on *Q* extended up to the sixth order. *c*′ is the anisotropic coupling factor between *Q* and *Φ*. *g* is the nonlinear coupling factor between mode K_3_ and mode $\Gamma_{2}^{-}$. *g*′ is the coupling factor between *Q* and *P*. *a_P_* is the self-energy factor of *P*. Coefficients $s_{Q}^{i}$ and $s_{P}^{i}$ scale the energy costs for the spatial variations of *Q* and *P*. They are defined as the stiffness parameters for *Q* and *P*, respectively.

Under the external stress–strain field, the interplay between the hexagonal lattice structure of the system and local stress/strain fields can drive the displacement of vortex cores within vortex domain structures. Starting from the interaction energy *f*_strain_ between the strain field and a vortex–antivortex pair, we transform its expression (6) from polar coordinates into Cartesian coordinates (7), as shown below.

(8) $$F_{\mathrm{int}}=\frac{\pi }{3}\lambda h\left[\left(\varepsilon_{xx}-\varepsilon_{yy}\right)\left(y_{A}-y_{V}\right)+2\varepsilon_{xy}\left(x_{A}-x_{V}\right)\right]$$

where (*x*_V_, *y*_V_) and (*x*_A_, *y*_A_) are the Cartesian coordinates of the vortex and antivortex, respectively. *ε_ij_* is the strain tensor. *λ* is the energy coupling coefficient, and *h* is the thickness of the sample.

By taking differential concerning their Cartesian coordinates, the force (termed the Magnus force, by analogy with fluid mechanics) acting on the vortex *f*_V_ and antivortex cores *f*_A_ is given by:

(9) $$f_{V}=-\frac{\partial F_{\mathrm{int}}}{\partial x_{V}}=-f_{A}=\frac{\pi }{3}\lambda h\left(2\varepsilon_{xy},\left(\varepsilon_{xx}-\varepsilon_{yy}\right)\right)$$

As illustrated in Figure 10c, the (anti)vortex cores move under the action of the Magnus force *f*_V_ (*f*_A_), resulting in the displacement of the domain walls that connect the vortex cores and separate the vortex domains. This process ultimately leads to the ordering of the vortex domain distribution. However, the intrinsic Young’s modulus of hexagonal manganite systems can be as high as 250 GPa [54,55]. Consequently, introducing stress at a single-crystal surface to generate a significant strain field remains a substantial experimental challenge. To address this issue, Wang et al. heated the single crystal to 1140 °C and subsequently introduced a stress–strain field by bending the sample using an aluminum rod. As a result, they successfully transformed the randomly distributed vortex domains in the ErMnO_3_ single crystal into a stripe-like configuration experimentally [91] (Figure 10c). The formation of stripe-like vortex domains occurs as the single crystal is slowly cooled across *T*_C_ from high temperature, during which the self-weight of the aluminum rod induces the formation and ordering of the vortex domains.

To further achieve localized and precise control over the distribution of ferroelectric vortex domains, Gao et al. employed a nanoindenter to apply a concentrated point load on the (001) plane at room temperature, thereby generating a residual strain field. Subsequently, during the high-temperature annealing process, the residual stress field induces the formation and ordering of the vortex domains [21]. Ultimately, a sixfold symmetric vortex domain structure is formed around the indentation created by the concentrated load, as shown in Figure 10d [21]. This phenomenon originates from the in-plane isotropy of the stress–strain field in the hexagonal lattice under a concentrated point load, while the Magnus force exhibits an alternating threefold symmetric distribution, with equal magnitudes but opposite signs for the vortex and antivortex cores. Under the action of the Magnus force, the vortex and antivortex cores migrate and accumulate along six symmetric axes, thereby transforming the initially random vortex domain configuration into a sixfold symmetric distribution. It is worth noting that this symmetric distribution is independent of the geometric symmetry of the indenter used to introduce the residual stress. Building upon the correlation between the in-plane concentrated point load and the sixfold symmetric distribution of vortex domains, moving the nanoindenter tip along a specific direction to create a nanoscratch naturally extends the range of the symmetric vortex domain arrangement from one dimension to two dimensions. Surprisingly, the vortex domain distribution induced by the nanoscratch is dependent on the scratching direction. Through the deliberate design of the indenter movement path, large-area, high-density, and parallelly aligned vortex domains with a single chirality (either vortex or antivortex) are formed between the antiparallel nanoscratches, as illustrated in Figure 10e. Moreover, the stripe-like vortex domains continue to conform to the topologically protected Z_2_ × Z_3_ six-domain structure. This observation appears intuitive, as the ferroelectricity in hexagonal manganite systems originates from structural distortions, and mechanical methods provide the most direct means of control. This work not only demonstrates the localized, precise, and designable control of topological ferroelectric vortex domains (topological defects) in hexagonal manganite systems but also provides a novel approach for manipulating domain structures in other structurally sensitive ferroic materials, particularly in bulk specimens.

## 6. Physical Properties for Potential Applications

### 6.1. Charged Domain Walls Under DC Bias

**Figure 11:**
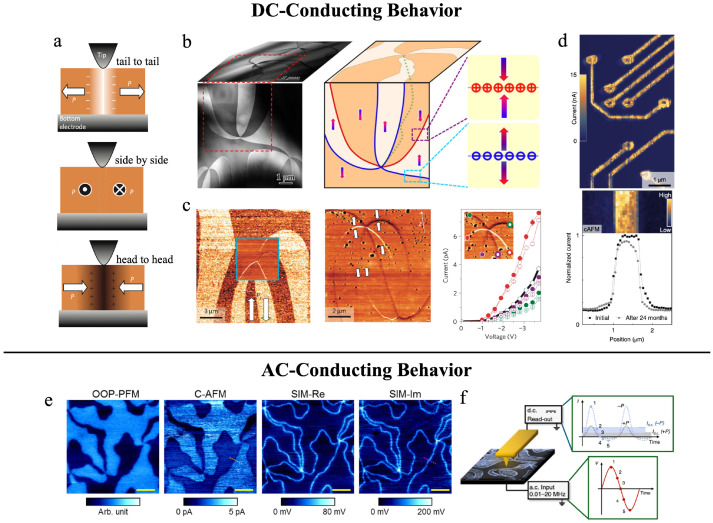
Charged domain walls in hexagonal manganites [80,81,82,103,149]. (**a**) Schematic illustration of tail-to-tail, side-by-side, and head-to-head domain walls; (**b**) Schematic diagram of vortex domains and charged domain wall distributions near the side-surface vortex cores; (**c**) Conductivity of charged domain walls near side-surface vortex cores. The dots in different colors represent the current values at the corresponding domain-wall positions shown in the inset; (**d**) insulator-to-conductor transition by oxygen vacancies influence; (**e**) “DC-Insulating & AC-Conducting” Behavior; (**f**) Half-Wave Rectification Effect. The numbers indicate the corresponding data signal points.

Considering that hexagonal manganite crystals exhibit a single polarization direction along the *c*-axis, and that the Z_2_ × Z_3_ vortex domains are randomly distributed and extend throughout the three-dimensional space of the entire single crystal. Therefore, on the side surfaces containing the *c*-axis (i.e., the *ac* and *bc* planes), non-neutral domain walls (charged domain walls) arise in the vicinity of the (anti)vortex cores due to the presence of a nonzero polarization component. Wu et al. and Meier et al. confirmed via cAFM that, on the side surfaces of HoMnO_3_ and ErMnO_3_ single crystals, three types of charged domain walls coexist near the vortex cores: head-to-head, side-by-side, and tail-to-tail configurations, as shown in Figure 11a–c [80,82]. Local current measurements indicate that the DC conductivity of different domain walls follows the order: *σ*_tail-to-tail_ > *σ*_side-by-side_ > *σ*_head-to-head_. This behavior arises because HoMnO_3_ and ErMnO_3_ single crystals are p-type semiconductors [47,67], where holes accumulate at negatively charged tail-to-tail domain walls, thereby enhancing their conductivity. Additionally, holes compensate the positive charge at head-to-head domain walls, thereby suppressing their conductivity. The coexistence of charged domain walls with different conductive states within a single crystal is exceedingly rare and differs from the unipolar conductive charged domain walls found in other systems, such as BiFeO_3_ [85,86,140], LiNbO_3_ [141,142], PbTiO_3_ [87,88], BaTiO_3_ [89], (Ca,Sr)_3_Ti_2_O_7_ [90], Sn_2_P_2_S_6_ [143,144], Bi_2_WO_6_ [145].

The anisotropic conductivity exhibited by ferroelectric topological defects in hexagonal manganites provides a platform for exploring the use of domain walls as electronic transport channels [46,80]. The three coexisting charged domain walls with distinct conductive states exhibit high stability due to topological protection [83,146]. Compared to magnetic skyrmions, ferroelectric topological defects offer several advantages. For instance, the domain walls in hexagonal manganites are approximately 7 Å wide, enabling ultra-high-density arrangements in three-dimensional space. Moreover, domain wall displacement is voltage-controlled and does not require current, which facilitates low-power operation and suppresses energy loss due to Joule heating. In addition, since the conductivity at charged domain walls arises from bound charges, electrostatic potential, and locally bent band structures, the carrier mobility is among the highest in oxide systems, making them promising candidates for novel information storage and processing devices, such as reservoir computing [7,103,127,139,147,148]. In addition to the intrinsic conductivity of charged domain walls driven by electrostatics, defect-activated extrinsic effects are widely present in the otherwise insulating domain walls of hexagonal manganites. Under the influence of local oxygen vacancies, the conductivity at domain walls can undergo an insulator-to-conductor transition (Figure 11d) [9]. Through the stoichiometric design of oxygen content, such behavior can be flexibly tuned for practical applications, enabling hexagonal manganites to function as sensors for oxygen and temperature.

### 6.2. Conductivity and Half-Wave Rectification Under AC Bias

Compared with DC conductivity, the side-by-side domain walls in hexagonal manganites exhibit higher conductivity under high-frequency (10^6^~10^10^ Hz) AC conditions, as illustrated in Figure 11e. The response of domain walls within the (001) plane under high-frequency AC is governed by the oscillation of bound charges rather than conduction by free carriers [79,147]. Due to differences in anisotropic conductivity and distinct conduction mechanisms, DC conduction occurs only within the *ac* or *bc* planes of hexagonal manganite single crystals, whereas AC conduction occurs within the *ab* plane. When an AC signal in the kilohertz-to-megahertz frequency range is applied, neutral domain walls in hexagonal manganites exhibit rectifying behavior (Figure 11f), enabling atomic-scale conversion from AC to DC [81,84]. Compared with conventional metal–semiconductor and semiconductor–semiconductor heterojunctions, the nanoscale diode effect at domain walls features an extremely small lateral dimension, defined by the minimal contact area of the probe tip (~1 nm^2^). Since the half-wave rectification effect originates from charge compensation induced by oxygen interstitial defects, the key parameters of domain-wall-based nanoscale diodes, such as the output frequency and amplitude of the AC signal, can be controlled via oxygen content. In this way, nanoscale electronics based on domain walls in hexagonal manganite single crystals can be extended to the AC regime, enabling the design of nanocapacitors, nanoinductors, and nanoscale transformers.

### 6.3. Dielectric and Ferroelectric Properties

**Figure 12:**
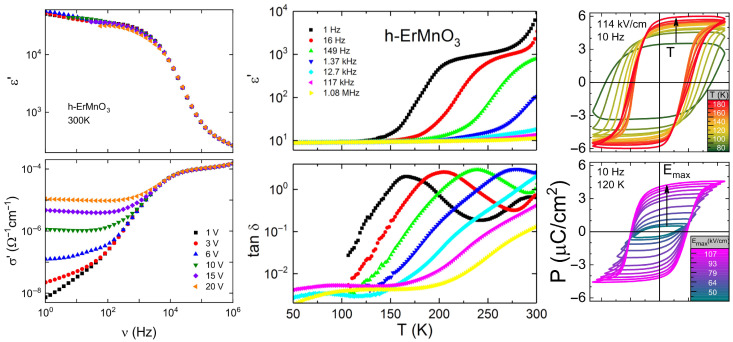
Dielectric spectra and ferroelectric hysteresis loops of hexagonal manganites [95,154].

The dielectric properties of ferroelectric materials represent the macroscopic manifestation of their intrinsic physical characteristics, including the behavior of ferroelectric domains and domain walls in charge accumulation, carrier transport, and conduction mechanisms. Moreover, these properties provide crucial insights into the potential practical applications of such materials. Researchers have conducted extensive investigations into the dielectric properties of hexagonal manganites. In 2011, Monika et al. identified two dielectric peaks and three distinct dielectric response mechanisms within 20~700 °C on YMnO_3_ [150]. In the range of 20~200 °C, the dielectric response was dominated by dipole hopping with an activation energy of 0.36 eV. Between 200~400 °C, electron hopping induced by the secondary ionization of oxygen vacancies became the predominant mechanism, characterized by an activation energy of 1.13 eV. At temperatures above 400 °C, DC conductivity made the major contribution. Ren et al. further investigated the nonlinear conductivity of YMnO_3_ ceramics annealed under different atmospheres across various temperature ranges [151]. To eliminate extrinsic factors such as grain and grain boundary effects, as well as to assess the influence of different electrode interfaces on surface barrier layers. Subsequent studies focused on the intrinsic dielectric properties of hexagonal manganites single crystals [95,152,153,154]. The intrinsic dielectric spectra and AC conductivity of hexagonal manganites are shown in Figure 12. The results revealed several intrinsic dielectric characteristics: (1) Domain walls act as internal barrier layers that induce additional dielectric relaxation; (2) Neutral domain walls exhibit higher conductivity than ferroelectric domains; (3) Ferroelectric domains display anisotropic dielectric responses. 

In the study of ferroelectricity, Ruff et al. investigated the temperature-, frequency-, and field-dependent ferroelectric properties of ErMnO_3_ single crystals through ferroelectric hysteresis loop measurements [95]. They found that leakage currents arising from interfacial polarization led to broadened hysteresis loops at elevated temperatures, with a notable frequency dependence, as shown in Figure 12. Moreover, the saturation polarization and coercive field of the ErMnO_3_ single crystal were determined to be 5~6 μC/cm^2^ and 40 kV/cm, respectively. The PUND-mode hysteresis loop measurements of YMnO_3_ single crystals [40] revealed comparable values of saturation polarization and coercive field to those of ErMnO_3_ [46].

### 6.4. Thermal Conductivity

In earlier studies employing the conventional steady-state technique, the thermal conductivity of ErMnO_3_ single crystals at low temperatures was investigated. By extrapolating the temperature-dependent thermal conductivity curve, the room-temperature thermal conductivity was estimated to be approximately 7 W·m^−1^·K^−1^ [155,156]. With the advent of advanced measurement techniques, it has become possible to accurately determine the intrinsic thermal conductivity of materials. In recent years, researchers have characterized the intrinsic thermal conductivity of hexagonal manganites using the time-domain thermo-reflectance (TDTR) technique. Owing to the symmetry of the hexagonal lattice, the thermal conductivity of hexagonal manganites exhibits pronounced anisotropy, with the out-of-plane and in-plane thermal conductivities measured to be 7.8 W·m^−1^·K^−1^ and 5.5 W·m^−1^·K^−1^, respectively [133].

**Figure 13:**
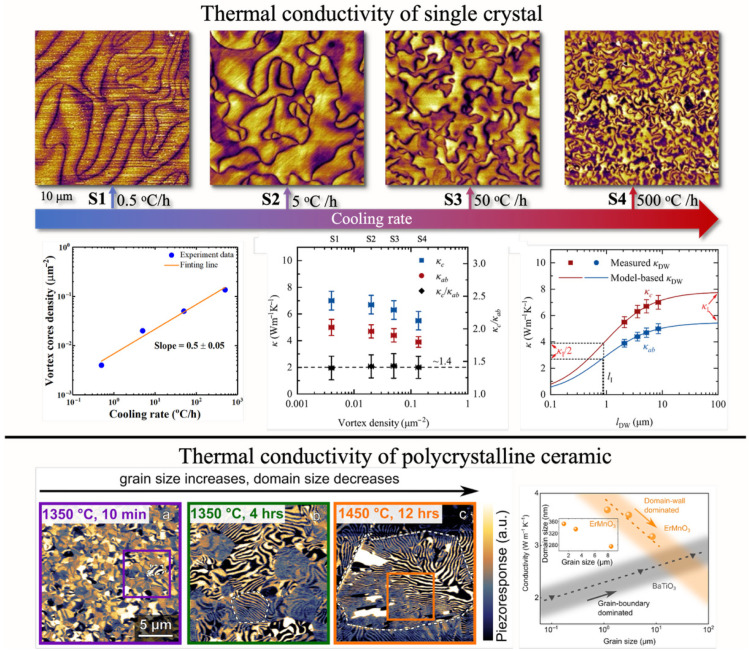
Thermal conductivity of hexagonal manganites [133,157].

Furthermore, it has been demonstrated that the density of topological defects can effectively modulate the thermal conductivity of hexagonal manganites. According to the Kibble–Zurek mechanism, varying the cooling rate during the annealing process can alter the topological defect density of ErMnO_3_ by up to two orders of magnitude, as shown in Figure 13. Consequently, both the out-of-plane and in-plane thermal conductivities at room temperature were found to decrease synchronously by approximately one-third, indicating that the increased domain wall density induced by a higher defect density significantly enhances phonon scattering at domain walls. In polycrystalline ceramic samples, additional grain boundary effects further strengthen phonon scattering, thereby reducing the thermal conductivity of hexagonal manganites to approximately 3 W·m^−1^·K^−1^ [157]. An anomalous size-dependent behavior was also observed, where the thermal conductivity decreases with increasing grain size. This counterintuitive relationship between heat transport and microstructure is attributed to phonon scattering at ferroelectric domain walls. In larger grains, a denser arrangement of domain walls reverses the conventional grain-boundary-dominated transport behavior.

## 7. Differences in Material Forms and Corresponding Properties

### 7.1. Preparation Methods of Single Crystal

Hexagonal manganite single crystals can be grown by several techniques, among which the floating-zone (FZ) method [158,159] and the high-temperature flux method [71,160] are the most widely employed. The FZ technique represents the state of the art for single-crystal growth: because the material neither contacts a crucible nor requires a flux during growth, the resulting crystals can be separated easily and are less susceptible to contamination. The high thermal gradient inherent to the FZ process enables rapid growth. In addition, the FZ method yields large, rod-shaped crystals that are convenient for subsequent processing, and targeted crystallographic orientations and dimensions can be obtained through post-growth cutting.

In contrast, the high-temperature flux method grows single crystals with nice facet and easy crystalline orientation than the FZ technique. For the growth of hexagonal manganite crystals, Bi_2_O_3_ can be used as an effective flux to obtain high-quality single crystals, and several practical strategies are available to overcome the challenge of separating the crystals from the flux. Although the relatively small thermal gradient in the flux-growth process slows the growth rate, it mitigates thermal-stress issues and thus allows for the preparation of large single crystals. However, due to the layered nature of the hexagonal manganite lattice (with large interplanar spacing along the c-axis), crystals grown by the flux method typically form as thin platelets, which poses challenges for subsequent machining and processing. Moreover, because the FZ growth temperature exceeds the Curie temperature of the hexagonal manganite family, the resulting domain configuration is invariably a vortex-type structure. Therefore, investigations of stripe domains, loop domains, and transitions among different domain structures require single crystals grown by the high-temperature flux method [71].

### 7.2. Differences in Properties Between Single Crystals and Thin Films

In addition to the intrinsic properties of hexagonal manganites obtained from single-crystal specimens that are the primary focus of this review, quite a few studies have investigated the properties of hexagonal manganite thin films. In contrast to single crystals, hexagonal manganite thin films rarely develop Z_2_ × Z_3_ vortex domain structures spontaneously. Such domain structures were not realized until Pang et al. achieved them via epitaxial growth on Al_2_O_3_ (0001) substrates [161,162]. However, substrate engineering can overcome the limitations imposed by intrinsic properties, enabling the emergence of novel phenomena, such as charge ordering [111], ferroelectric photovoltaics [163], Giant Flexoelectric Effect [164]. Moreover, lattice matching or mismatch in epitaxial thin films not only determines the crystallographic orientation but also induces structural distortions, which in turn strongly influence ferroelectric polarization and domain configurations. In particular, in hexagonal manganite systems, the trimerization distortion (K_3_ mode) associated with the primary lattice instability is intimately coupled to the mechanism of polarization formation. This implies that epitaxial constraint and interfacial strain can directly modulate both the transition temperature and the magnitude of ferroelectricity. As the film thickness is reduced, the ferroelectric transition temperature *T*_C_ progressively decreases. Recently, Nordlander et al. inserted a highly conductive indium tin oxide (ITO) layer between the substrate and the YMnO_3_ film. This approach not only mitigated lattice-mismatch-induced dislocations but also preserved the high crystalline quality and surface smoothness of the film, thereby establishing a viable technological pathway for studies of hexagonal manganite-based multiferroic heterostructures [165]. With regard to mechanical properties, epitaxial thin films [54] of hexagonal manganites exhibit mechanical behavior that is largely comparable to that of their bulk counterparts [55]. This similarity is likely associated with the intrinsic layered nature of the hexagonal lattice, which gives rise to a quasi-two-dimensional structural framework.

In summary, the properties of hexagonal manganite thin films depend strongly on substrate selection and strain accommodation, which is closely related to the fact that ferroelectricity in hexagonal manganites originates from structural distortions. In particular, in contrast to conventional proper ferroelectrics, the ferroelectric behavior of hexagonal manganite thin films is governed more by epitaxial engineering than by a simple thickness limit. By tailoring chemical composition, strain state, interfacial configuration, and buffer-layer design through epitaxial approaches, it is possible to modulate the stability of the ferroelectric phase, the magnitude of polarization, domain architectures, and even multiferroic coupling phenomena. For a more comprehensive discussion of hexagonal manganite thin films, the reader is referred to existing review articles [166]. In comparison with epitaxial hexagonal manganite thin films, which require stringent growth conditions, recently reported approaches such as the spray pyrolysis method enable the fabrication of polycrystalline thin films in a much more straightforward manner. These simplified synthesis routes provide a practical platform for property investigations based on polycrystalline samples, for example, studies of ferroelectric photovoltaic effects associated with narrow band-gap characteristics [167]. In addition to polycrystalline thin films, ceramics represent another form of polycrystalline samples that are well suited for investigating dielectric properties associated with grain boundaries and domain walls in hexagonal manganites [95,150,151,152,153,154]. However, owing to their relatively large leakage currents and low saturated polarization, hexagonal manganites are not suitable as ceramic dielectric energy-storage materials.

## 8. Summary and Outlook

### 8.1. Summary

This review focuses on ferroelectric topological defects in hexagonal manganites, providing a comprehensive summary of the system’s ferroelectric origins, the formation and evolution of ferroelectric domain structures, the external-field modulation of topological defects, and associated advanced physical phenomena. In hexagonal manganites, the regulation of topological defects fundamentally arises from the interplay between external physical fields (such as electric, thermal, and mechanical fields) and the hexagonal lattice, encompassing rich mechanisms of energy competition and interdependent interactions. The formation, distribution, and evolution of the unique ferroelectric domain structures in hexagonal manganites are profoundly influenced by these external fields, giving rise to numerous novel physical effects and offering a promising platform for next-generation micro- and nanoelectronic devices. As a representative ferroelectric material, hexagonal manganites exhibit abundant physical mechanisms and broad application potential, which not only enrich the fundamental understanding of ferroelectric domain physics but also provide valuable insights for research on other ferroic materials.

### 8.2. Outlook

#### 8.2.1. Effective Manipulation of Ferroelectric Topological Defects

The macroscopic ferroelectricity of ferroelectric materials originates from the polarization switching of internal ferroelectric domains and the motion of domain walls. In the context of next-generation micro- and nanoelectronic device applications, achieving effective regulation of ferroelectric domains, domain walls, and other topological defects represents a key scientific challenge. Current approaches for controlling topological defects in hexagonal manganites primarily focus on external electric fields, thermal annealing processes, and mechanical loading. However, each of these methods presents inherent limitations: electric-field control cannot displace the vortex core; modulation of annealing and cooling rates fails to achieve localized and precise control; and mechanical manipulation tends to be destructive. Therefore, optimizing existing control strategies and exploring novel regulation methods constitute the future directions for research on the modulation of ferroelectric topological defects.

The formation of different ferroelectric domain structures in hexagonal manganites is closely related to the single-crystal growth temperature and subsequent thermal treatment [75,76,77]. Naturally grown hexagonal manganite single crystals typically exhibit stripe-like domain structures, whereas vortex domain structures can emerge after annealing above the Curie temperature. In terms of spatial distribution, it has been observed that the ferroelectric domain structures gradually evolve from the crystal surface toward the bulk interior [71], suggesting that the formation of ferroelectric topological defects may be influenced by boundary effects. Furthermore, the transition from vortex to stripe domains observed in as-grown single crystals implies that the formation of ferroelectric topological defects is also affected by spontaneous residual strain [70]. When subjected to external pressure, the distribution of ferroelectric topological defects in ErMnO_3_ single crystals is found to change correspondingly [137,138]. Collectively, these findings indicate that the formation of ferroelectric topological defects in hexagonal manganites is strongly influenced by boundary effects, temperature fields, and stress/strain fields, and thus warrants further in-depth investigation to elucidate the underlying mechanisms.

#### 8.2.2. The Relationships Between Topological Defects and Macroscopic Physical Properties

The ferroelectric topological defects in hexagonal manganites exhibit anisotropic conductivity, providing a research platform for exploring domain walls as potential electronic transport channels [46,80]. In the vicinity of ferroelectric topological defects, three distinct types of charged domain walls with different conductive states can coexist simultaneously [83], and their stability is ensured by topological protection [146]. Compared with magnetic skyrmions, ferroelectric topological defects possess several advantages. For instance, the domain wall width in hexagonal manganites is approximately 7 Å, enabling ultra-high-density arrangements in three-dimensional space. Furthermore, domain wall displacement can be controlled purely by an applied voltage without the need for electrical current, which contributes to low power consumption and mitigates energy loss due to Joule heating. In addition, the conductivity at charged domain walls originates from bound charges, electrostatic potentials, and locally bent band structures, resulting in carrier mobilities among the highest in oxide systems. These features make them promising candidates for novel information storage and processing devices [7,103,127,139,147]. In particular, hexagonal manganite system has been proposed as an excellent playground for investigating reservoir computing concept [127], owing to polarization enabled nonlinearity, reversible long range domain wall motion [64] that given shot term fading memory, together with complexity. Beyond the intrinsic conductivity arising from electrostatically driven charged domain walls, non-intrinsic effects activated by defects are also prevalent in non-conductive domain walls of hexagonal manganites. Under the influence of local oxygen vacancies, the conductivity at domain walls can undergo an “insulator-to-conductor” transition. By designing nonstoichiometric oxygen compositions, their functional properties can be flexibly tuned, enabling applications of hexagonal manganites as oxygen and temperature sensors [9]. Compared with conventional “metal–semiconductor” and “semiconductor–semiconductor” heterojunctions, the nanoscale diode effect at domain walls exhibits the smallest possible lateral dimension (defined by the minimal tip–sample contact area of approximately 1 nm^2^). Consequently, the half-wave rectification effect in hexagonal manganites, originating from charge compensation behaviors induced by interstitial oxygen defects, allows the key parameters of domain-wall-based nanodiodes to be tuned via oxygen content, such as the frequency and amplitude of alternating current output. This advancement extends domain-wall-based nanoelectronics in hexagonal manganites into the AC regime, paving the way for the design of nanoscale capacitors, inductors, and transformers. Given such promising prospects, elucidating and establishing the structure–property relationships between the microscopic domain configurations and macroscopic physical properties of hexagonal manganites represent critical and compelling scientific challenges.

## Figures and Tables

**Table 1:** Summary of the intrinsic mechanical parameters on the (001) plane of hexagonal manganites.

	Young’s Modulus (GPa)				Hardness (GPa)	Fracture Toughness (MPa·m^1/2^)
	CR-AFM	Nanoindentation		DFT	Nanoindentation	Nanoindentation
		10 mN Loading	450 mN Loading			
LuMnO_3_	260.3	294	243	246	11.85	3.6
ErMnO_3_	258.7	244	126	231	10.90	2.0
YMnO_3_	254.7	225	106	216	11.29	0.9

**Table 2:** Techniques for observation ferroelectric domain of *h*-RMnO_3_ in real space.

Technique		Resolution	Physical Features
Optical microscopy	Chemical etching	cm~μm	topography; ferroelectric domains; the transition between Type I and II
Scanning probe microscopy	PFM	μm~100 nm	Electromechanical response; polar domains
	EFM	μm~100 nm	potential gradient; domain wall hall conductance by incorporating magnetic field
	cAFM	μm~100 nm	DC conductance or tunnelling current; charged domain boundaries; oxygen vacancy migration
	sMIM	μm~50 nm	domain and domain Walls; AC electrical conductance in the gigahertz range
Electron Microscopy	Dark Field TEM	μm~nm	domains and domain walls (including antiphase boundaries)
	HAADF-STEM	nm~pm	atomical structural distortions of domains and domain boundaries
	SEM	mm~nm	contrast of domain and domain wall; out-of-plane and in-plane polarization

## Data Availability

No new data were created or analyzed in this study. Data sharing is not applicable to this article.
